# Supplementary material for: Protein quality control and regulated proteolysis in the genome‐reduced organism Mycoplasma pneumoniae
Source: Mol Syst Biol. 2020 Dec 15;16(12):e9530. doi: 10.15252/msb.20209530 (PMC7737663; doi:10.15252/msb.20209530)
Supplement: Supplementary file 1 — Appendix [file MSB-16-e9530-s001.pdf]

Appendix

Table of contents

Appendix Figure S1. Construction of Lon and FtsH conditional mutants in *M. pneumoniae.*

Appendix Figure S2. Growth curve analysis for ΔIndLon, ΔIndFtsH, and ΔIndLon_FtsH strains.

Appendix Figure S3. Dot blot analysis of cellular DNA replication.

Appendix Figure S4. Volcano plots of the differences in protein abundance between inducing and depleting conditions.

Appendix Figure S5. Volcano plots of the differences in mRNA levels between inducing and depleting conditions.

Appendix Figure S6. Immunoblot analyses of selected Lon and FtsH candidate substrates.

Appendix Figure S7. Immunoblot analyses of selected FtsH candidate substrates.

Appendix Figure S8. Analysis of degron motif properties.

Appendix Figure S9. Sequence alignment of HsdS subunits present in *M. pneumoniae.*

Appendix Figure S10. Mutational analysis to identify Lon degrons in MPN201 (HsdS).

Appendix Figure S11. Mutational analysis to identify Lon degrons in MPN304 (ArcA Nt).

Appendix Figure S12. Correlation between proteome changes in responses to Lon and FtsH depletions.

Appendix Figure S13. Fold change distribution of Lon candidate substrates in Triton X-100 insoluble fractions of cells grown under Lon inducing conditions.

Appendix Figure S14. Transposon mutagenesis screening to identify putative gene mutations suppressing lethality associated with Lon and FtsH depletion.

Appendix Table S1. Mycoplasma strains used in this study.

Appendix Table S2. Gibson cloning strategy for each plasmid constructed in this study.

Appendix Table S3. Primers used in this study.

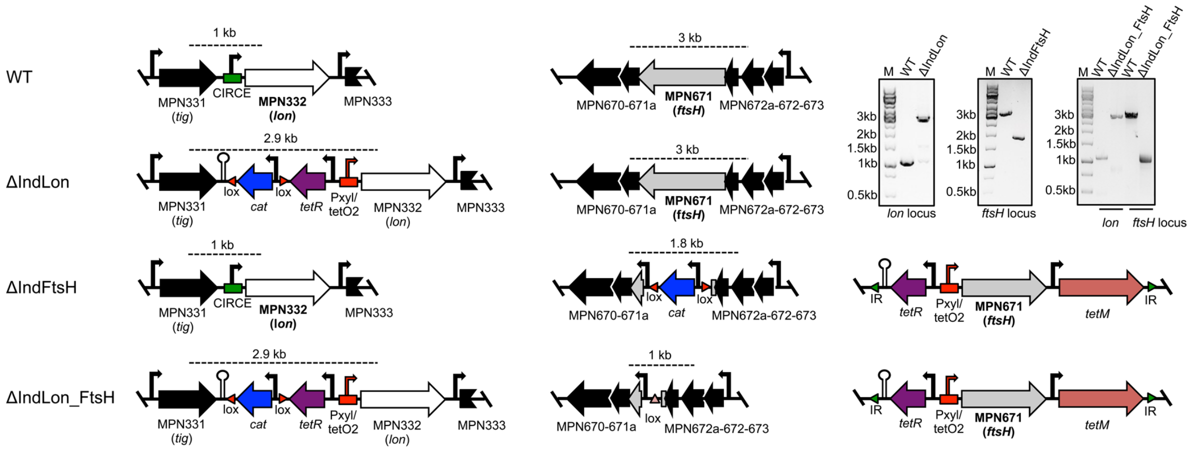

Appendix Figure S1. Construction of Lon and FtsH conditional mutants in *M. pneumoniae.*
Schematic representation of the genetic architecture after genome edition of ΔIndLon, ΔIndFtsH and ΔIndLon_FtsH conditional mutants as compared to the wild-type (WT) strain. The DNA rearrangement in the *lon* and *ftsH* locus is shown for each strain. The *ftsH* inducible platform inserted by transposon delivery is also shown for ΔIndFtsH and ΔIndLon_FtsH strains. The Pxyl/tetO2 inducible promoter is highlighted with a red bent arrow and the terminator sequence used to isolate the promoter is represented by a hairpin structure. The *tetR* repressor gene and the resistance markers *cat* and *tetM* are indicated in purple, blue and red, respectively. The CIRCE regulatory element replaced by the *lon* inducible platform is shown in green. Agarose electrophoresis gels showing the PCR screening confirmed the intended genome rearrangements at the *lon* and *ftsH* locus (top right panel). The PCR products and expected sizes for each strain are shown in the scheme.

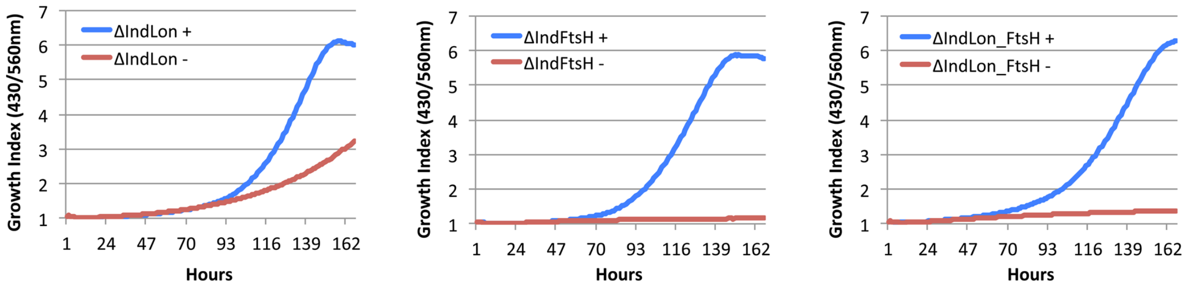

Appendix Figure S2. Growth curve analysis for ΔIndLon, ΔIndFtsH, and ΔIndLon_FtsH strains grown under inducing (+) or depleting (–) conditions as determined by the 430/560 absorbance rate index that shows pH changes in the medium.

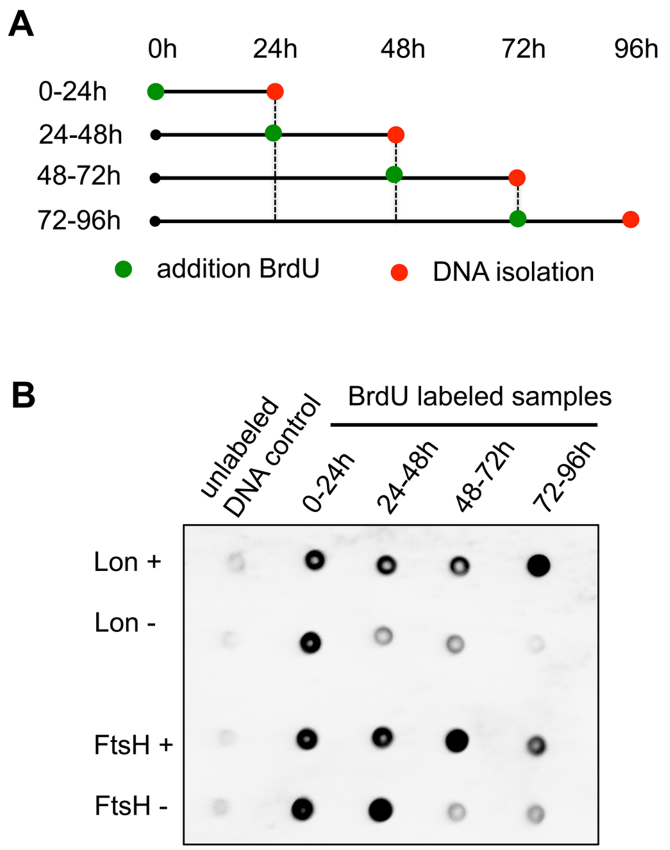

Appendix Figure S3. Dot blot analysis of cellular DNA replication.

(A). ΔIndLon and ΔIndFtsH strains grown under inducing (+) or depleting (–) conditions were incubated with 100µM BrdU during 24h at different interval time points across the growth curve. After BrdU labeling, BrdU pulse-labeled cells were collected and total DNA extracted.

(B). Dot blot DNA hybridization analysis of BrdU labeled samples indicated in panel A. A DNA unlabeled control was also included. A total of 100 ng of DNA was denatured and spotted on a Hybond-N+ membrane. Following UV cross-linking, labeled DNA was visualized using an anti-BrdU antibody. BrdU incorporation was reduced after 24h and 48h of depletion of Lon and FtsH, respectively. A representative experiment is shown.

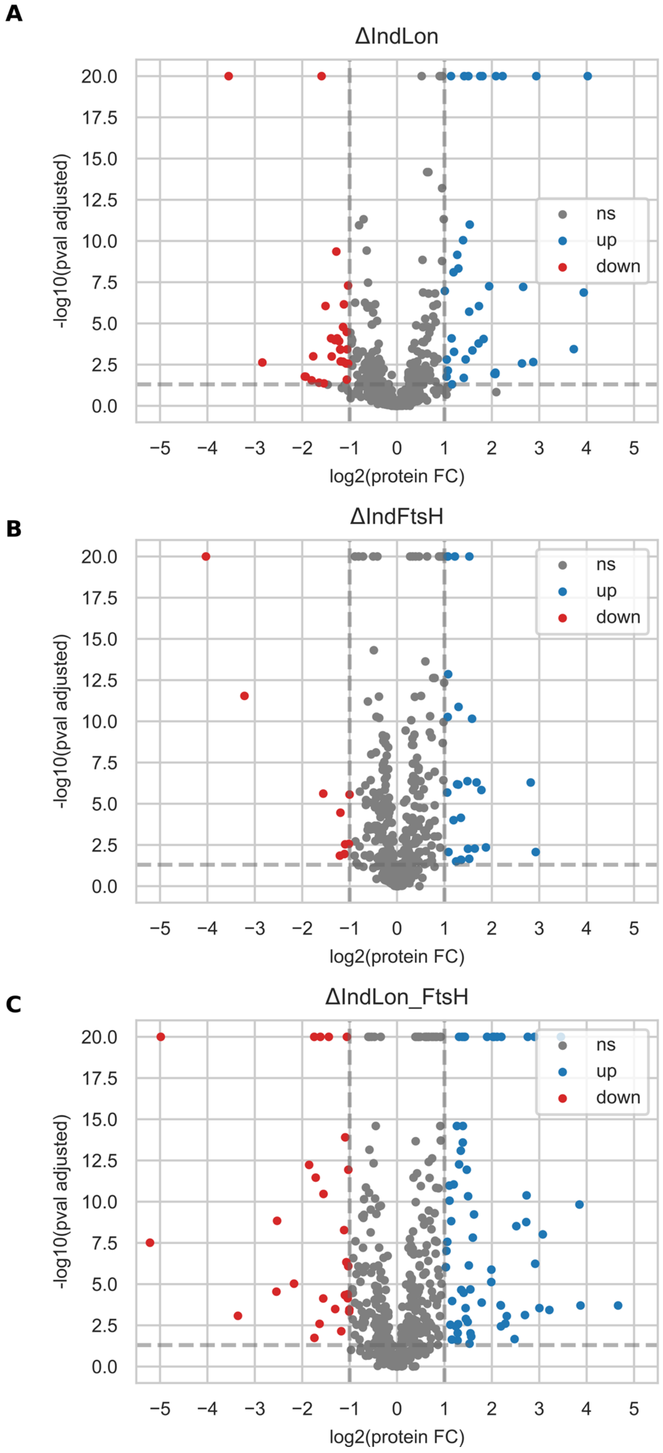

Appendix Figure S4. Volcano plots of the differences in protein abundance between inducing and depleting conditions. Differences in protein abundance between inducing (+) and depleting (–) conditions (48h and 72h of depletion for Lon and FtsH, respectively) are reported as $\log_2$ of fold-changes for conditional mutants ΔIndLon **(A)**, ΔIndFtsH **(B)** and ΔIndLon_FtsH **(C)**. Statistical significance, calculated following a peptide-based statistical method, is reported as $-\log_{10}$ of the adjusted p-value after multiple tests correction. Significantly regulated proteins were defined based on a false discovery rate (FDR) of 5% (dashed horizontal line) and a minimum fold change cut-off of 2 (dashed vertical lines). For better visualization, very small p-values were clipped at 1×10^{-20}.

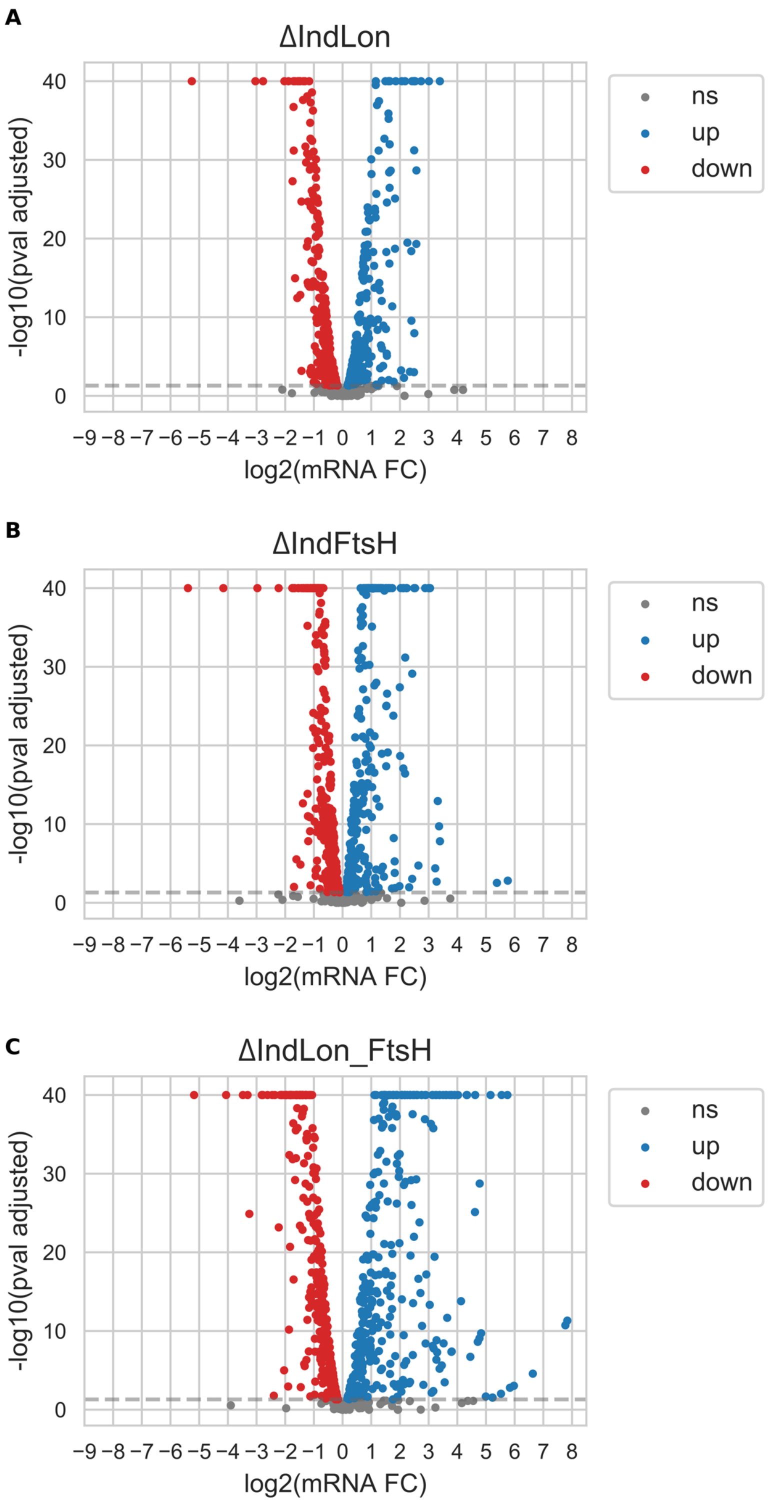

Appendix Figure S5. Volcano plots of the differences in mRNA levels between inducing and depleting conditions. Differences in mRNA levels between inducing (+) and depleting (–) conditions (48h and 72h of depletion for Lon and FtsH, respectively) are reported as $\log_2$ of fold changes for conditional mutants ΔIndLon **(A)**, ΔIndFtsH **(B)** and ΔIndLon_FtsH **(C)**. Statistical significance is reported as $-\log_{10}$ of the adjusted p-value after multiple tests correction. Significantly regulated genes were defined based on a false discovery rate (FDR) of 5% (dashed horizontal line). For better visualization, very small p-value were clipped at 1 x 10^{-40}.

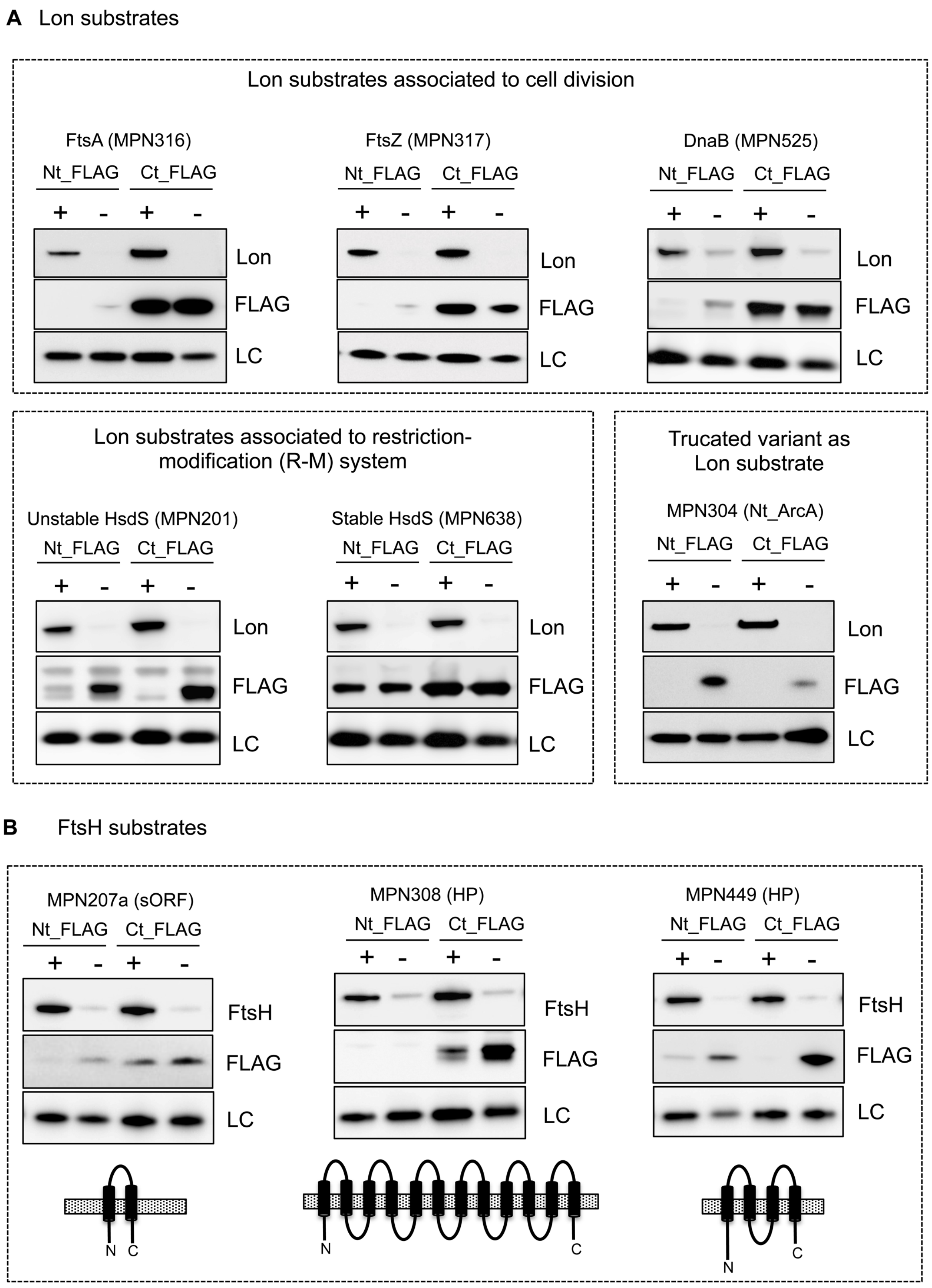

Appendix Figure S6. Immunoblot analyses of selected Lon and FtsH candidate substrates. **(A)** Lon candidate substrates. **(Upper panel)** N- and C-terminal FLAG-tagged derivatives of candidates substrates associated to cell division, including FtsA (MPN316), FtsZ (MPN317) and DnaB (MPN525) were expressed in the ΔIndLon mutant. Protein levels of Lon and candidate

substrates were then assessed by immunoblot under inducing (+) or depleting (–) conditions (48h of depletion) using anti-Lon and anti-FLAG antibodies. LC, loading control.

(Lower, left panel) Similar to the upper panel A, but for candidate substrates associated to the R-M system. Protein expression assessed by immunoblot of FLAG-tagged derivatives of MPN201 and MPN638 are shown as representatives of unstable and stable HsdS subunits, respectively. **(Lower, right panel)** Similar to the upper panel A, but showing an example of a truncated variant as a Lon candidate substrate. Specifically, protein expression assessed by immunoblot of FLAG-tagged derivatives of MPN304 (ArcA-Nt) that encodes the N-terminal fragment of an ArcA truncated variant is shown.

(B) FtsH candidate substrates. N- and C-terminal FLAG-tagged derivatives of MPN207a, MPN308 and MPN449 were expressed in the ΔIndFtsH mutant. Protein levels of FtsH and candidate substrates were then assessed by immunoblot under inducing (+) or depleting (–) conditions (72h of depletion) using anti-FtsH and anti-FLAG antibodies. For each protein, a schematic representation of the predicted transmembrane domains is also shown below.

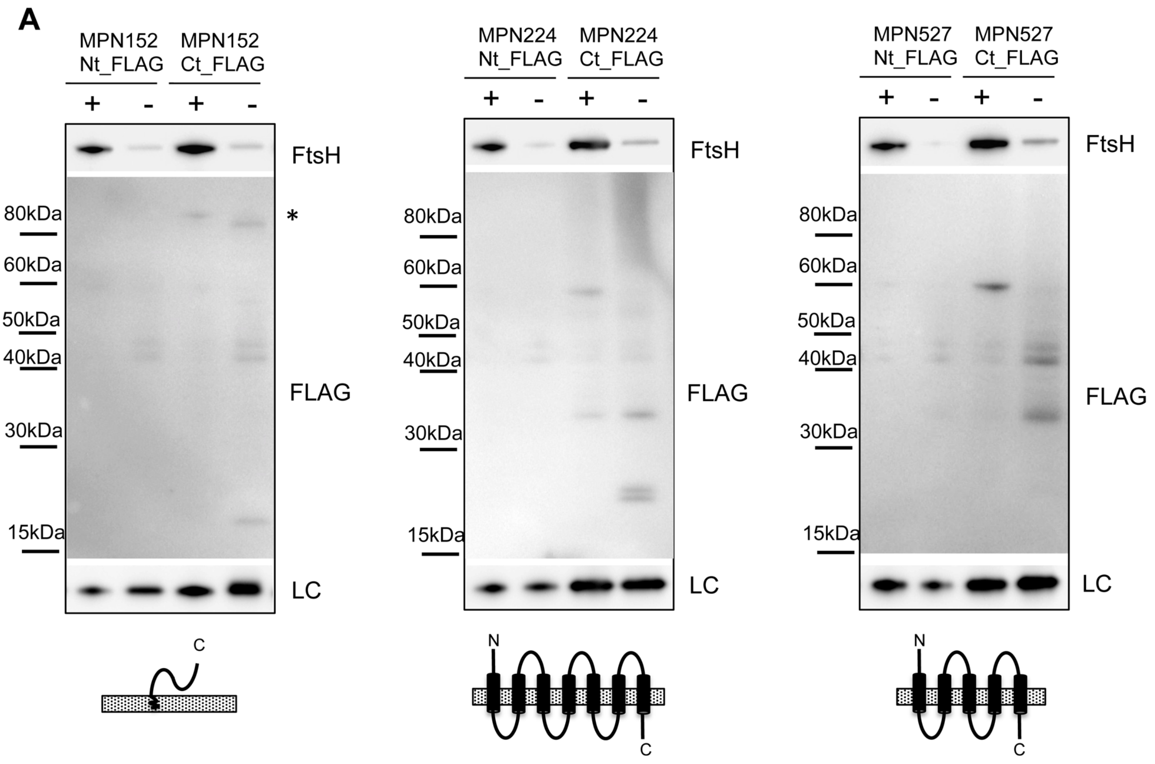

B

Candidate	copies / cell		Signal peptide	Annotation	Predicted size
	ΔIndFtsH +	ΔIndFtsH -			
MPN152	6	26	lipobox	lipoprotein	87.3 kDa
MPN207a	0	343	No	sORF	10.8 kDa
MPN224	48	107	No	lgt	42.8 kDa
MPN308	53	108	No	polyamine antiporter	62.1 kDa
MPN449	58	223	No	hypothetical protein	49.2 kDa
MPN527	0	15	No	hypothetical protein	24.7 kDa

Appendix Figure S7. Immunoblot analyses of selected FtsH candidate substrates.

(A). N- and C-terminal FLAG-tagged derivatives of candidate substrates were expressed in the ΔIndFtsH mutant (see also Fig. 4). Protein levels of FtsH and the candidate substrates were then assessed by immunoblot analysis during inducing (+) or depleting (–) conditions (72h of depletion) using anti-FtsH and anti-FLAG antibodies. LC, loading control. The asterisk indicates the predicted position of the detected protein. For each protein, a schematic representation of the predicted transmembrane domains is also shown below.

(B). Protein features and estimated protein copy numbers of selected FtsH candidate substrates of ΔIndFtsH mutant grown in inducing (+) or depleting (–) conditions.

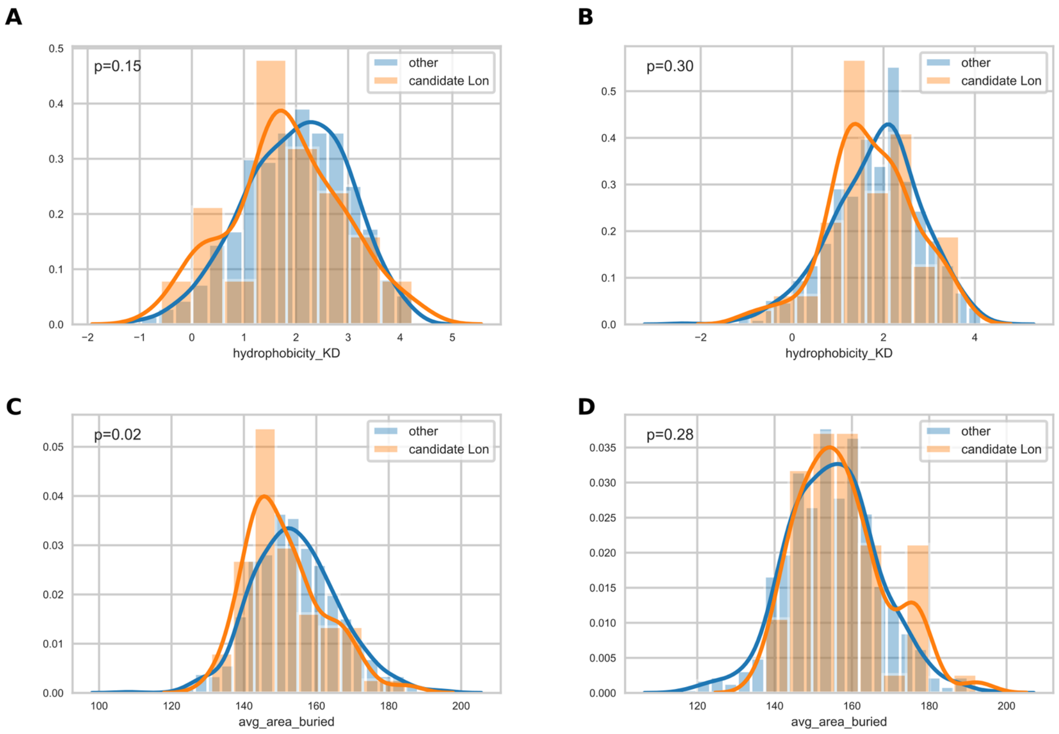

Appendix Figure S8. Analysis of degron motif properties.

Protein sequences at N- (panels **A** and **C**) and C-termini (panels **B** and **D**) of Lon candidate substrates were analyzed for putative signal in amino acid properties, as for example enrichment of hydrophobic region (panels A and B, Kyte-Doolittle hydrophobicity scale) and the average buried area (panels C and D). The terminus region of protein sequence was selected (25 residues). The average amino acid property score was computed over a rolling window of size 5. The maximum score was selected as representative of the most extreme signal in the terminus score. Distribution of the maximum score for substrate proteins did not show any significant difference (M.W.W. two-sided test) when compared to the background proteins (neither substrate of Lon nor of FtsH). A similar analysis of the average score over the last (first) 5 or 10 amino acids of protein sequences did not yield any significant enrichment.

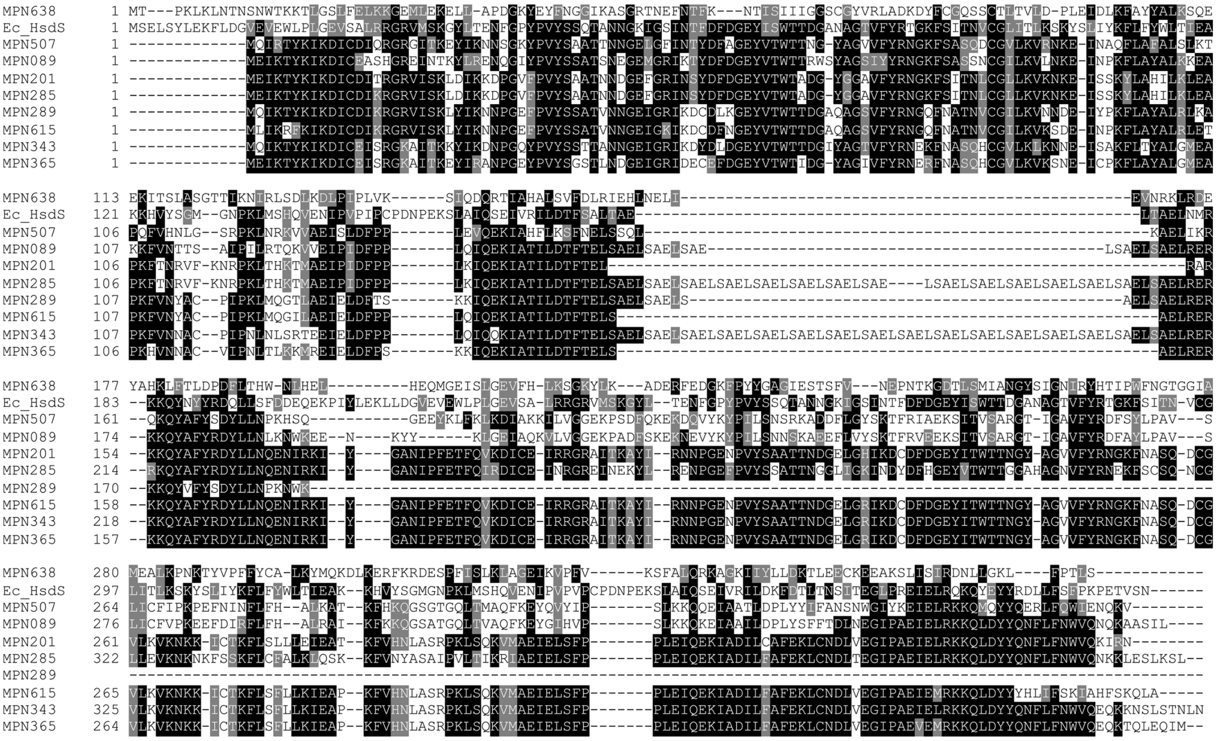

Appendix Figure S9. Sequence alignment of HsdS subunits present in *M. pneumoniae* compared to *E. coli* HsdS (WP_096840861.1). The structure of the orthologous protein of MPN638 (HsdS) in *M. genitalium* (MG438) has been solved (PDB identifier: 1YDX). The protein sequence of MPN638 differs from the other HsdS subunits, although it is similar to other HsdS at the structural level (Calisto et al., 2005).

Calisto BM, Pich OQ, Piñol J, Fita I, Querol E & Carpena X (2005) Crystal structure of a putative type I restriction-modification S subunit from Mycoplasma genitalium. J. Mol. Biol. 351: 749–762

A

\>MPN201
ME**IKTYKIKDICDI**TRGRVISKLDIKKDPG**VFPVY**SAATNNDGEFGRINS**YDFDGEYVTW**TADGYGGAVFYRNGKFSITNLCGLLKVKNKEISSKYLAHIL
KLEAPKFTNRVFKNRPKLTHKTMAEIPIDFPPLKIQEKIATILDTFTELRARKKQYAFYRDYLLNQENIRKIYGANIPFETFQVKDICEIRRGRAITKAYI
RNNPGENPVYSAATTNDGELGHIKDCDFDGEYITWTTNGYAGVVFYRNGKFNASQDCGVLKVKNKKICTKFLSLLLEIEATKFVHNLASRPKLSQKVMAEI
ELSFPPLEIQEKIADILCAFEKLCNDLVEGIPAEIELRKKQLD**YYQNFLFNWV**QKIRN

\>MPN201mut1(CtMPN638)
MEIKTYKIKDICDITRGRVISKLDIKKDPGVFPVYSAATNNDGEFGRINSYDFDGEYVTWTADGYGGAVFYRNGKFSITNLCGLLKVKNKEISSKYLAHIL
KLEAPKFTNRVFKNRPKLTHKTMAEIPIDFPPLKIQEKIATILDTFTELRARKKQYAFYRDYLLNQENIRKIYGANIPFETFQVKDICEIRRGRAITKAYI
RNNPGENPVYSAATTNDGELGHIKDCDFDGEYITWTTNGYAGVVFYRNGKFNASQDCGVLKVKNKKICTKFLSLLLEIEATKFVHNLASRPKLSQKVMAEI
ELSFPPLEIQEKIADILCAFEKLCNDLVEGIPAEIELRKKQLD**SIRDNLLGKLFPTLS**

\>MPN201mut2
ME**GKTDKGKDGCDG**TRGRVISKLDIKKDPGVFPVYSAATNNDGEFGRINSYDFDGEYVTWTADGYGGAVFYRNGKFSITNLCGLLKVKNKEISSKYLAHIL
KLEAPKFTNRVFKNRPKLTHKTMAEIPIDFPPLKIQEKIATILDTFTELRARKKQYAFYRDYLLNQENIRKIYGANIPFETFQVKDICEIRRGRAITKAYI
RNNPGENPVYSAATTNDGELGHIKDCDFDGEYITWTTNGYAGVVFYRNGKFNASQDCGVLKVKNKKICTKFLSLLLEIEATKFVHNLASRPKLSQKVMAEI
ELSFPPLEIQEKIADILCAFEKLCNDLVEGIPAEIELRKKQLDYYQNFLFNWVQKIRN

\>MPN201mut3
MEIKTYKIKDICDITRGRVISKLDIKKDPG**DGPDG**SAATNNDGEFGRINSYDFDGEYVTWTADGYGGAVFYRNGKFSITNLCGLLKVKNKEISSKYLAHIL
KLEAPKFTNRVFKNRPKLTHKTMAEIPIDFPPLKIQEKIATILDTFTELRARKKQYAFYRDYLLNQENIRKIYGANIPFETFQVKDICEIRRGRAITKAYI
RNNPGENPVYSAATTNDGELGHIKDCDFDGEYITWTTNGYAGVVFYRNGKFNASQDCGVLKVKNKKICTKFLSLLLEIEATKFVHNLASRPKLSQKVMAEI
ELSFPPLEIQEKIADILCAFEKLCNDLVEGIPAEIELRKKQLDYYQNFLFNWVQKIRN

\>MPN201mut4
MEIKTYKIKDICDITRGRVISKLDIKKDPGVFPVYSAATNNDGEFGRINS**GDGDGEDGTD**TADGYGGAVFYRNGKFSITNLCGLLKVKNKEISSKYLAHIL
KLEAPKFTNRVFKNRPKLTHKTMAEIPIDFPPLKIQEKIATILDTFTELRARKKQYAFYRDYLLNQENIRKIYGANIPFETFQVKDICEIRRGRAITKAYI
RNNPGENPVYSAATTNDGELGHIKDCDFDGEYITWTTNGYAGVVFYRNGKFNASQDCGVLKVKNKKICTKFLSLLLEIEATKFVHNLASRPKLSQKVMAEI
ELSFPPLEIQEKIADILCAFEKLCNDLVEGIPAEIELRKKQLDYYQNFLFNWVQKIRN

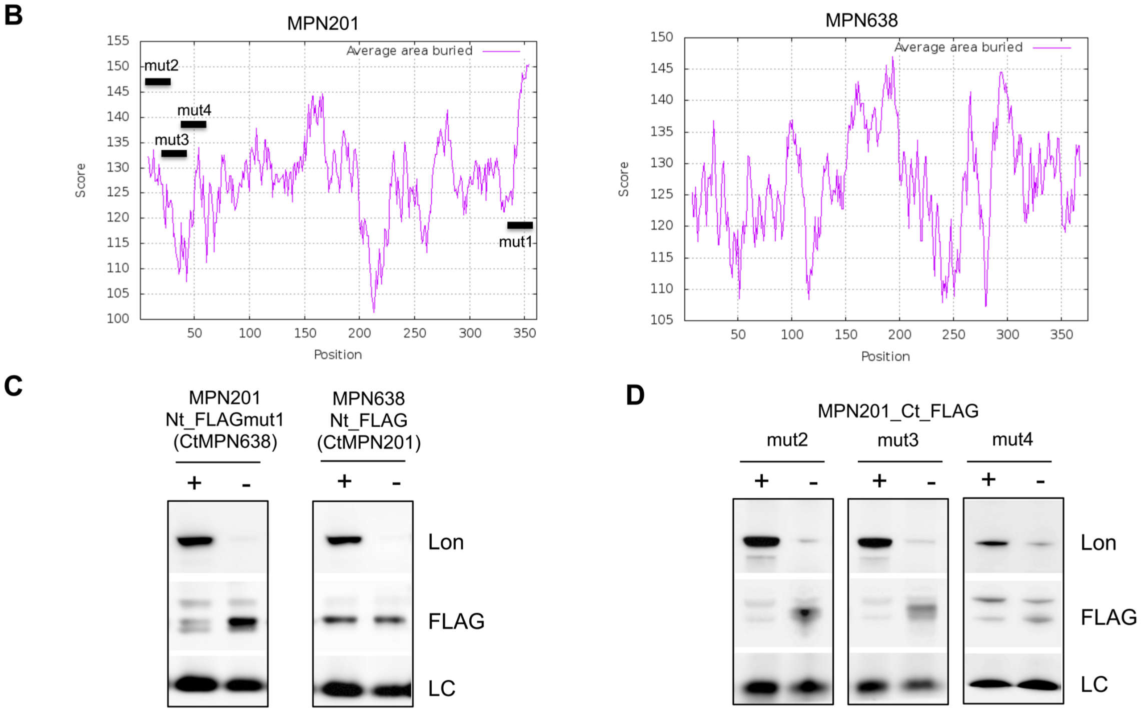

Appendix Figure S10. Mutational analysis to identify Lon degrons in MPN201 (HsdS).

(A) Amino acid sequence of MPN201and their mutant derivatives. Hydrophobic motifs that can potentially act as Lon degrons are highlighted in bold. Mutations performed in these putative Lon degrons are shown in red.

(B) Sequence analysis showing the surface-area burial score along MPN201 (HsdS) and MPN638 (HsdS) protein sequence. Analysis was performed using the ProtScale Tool selecting the “average area buried” amino acid scale (Gasteiger et al, 2005). Mutated regions in MPN201 are indicated in the plot.

(C) Protein stability assessment of MPN201 and MPN638 N-terminal FLAG-tagged derivatives in which their C-terminal regions were swapped and expressed in the ΔIndLon mutant. Protein levels were then determined by immunoblot using anti-Lon and anti-FLAG antibodies comparing inducing (+) or depleting (–) conditions (48h of depletion). LC, loading control.
(D) Protein stability assessment of MPN201 containing mutations in putative Lon degrons. C-terminal FLAG-tagged derivatives with mutations shown in panel A were expressed in the ΔIndLon mutant and protein levels determined by immunoblot as in panel C.

We hypothesized that the C-terminal hydrophobic motif YYQNFLFNWV could be responsible for Lon-mediated degradation, as this motif is mainly conserved in the unstable HsdS, but absent from MPN638 (Appendix Figure S9). To test this, we swapped the C-terminal regions of MPN201 and MPN638 and we assessed the stability of these proteins in the presence of Lon. We found that MPN201 was still degraded, whereas MPN638 remained stable (Appendix Figure S10C). We then searched for sequence motifs in MPN201 with a high score for buried surface-area, as these regions have been shown to correlate well in *E. coli* with Lon-dependent degradation (Gur & Sauer, 2008). As the N-terminal FLAG fusion slightly improved the stability of MPN201 (Appendix Figure S6), we focused particularly on motifs present in the N-terminal region. However, after several mutational analyses in candidate motifs, we were unable to identify a clear degradation signal in MPN201 (Appendix Figure S10D), suggesting the existence of multiple exposed degrons.

Gasteiger E., Hoogland C., Gattiker A., Duvaud S., Wilkins M.R., Appel R.D., Bairoch A.; *Protein Identification and Analysis Tools on the ExPASy Server;* (In) John M. Walker (ed): The Proteomics Protocols Handbook, Humana Press (2005). pp. 571-607

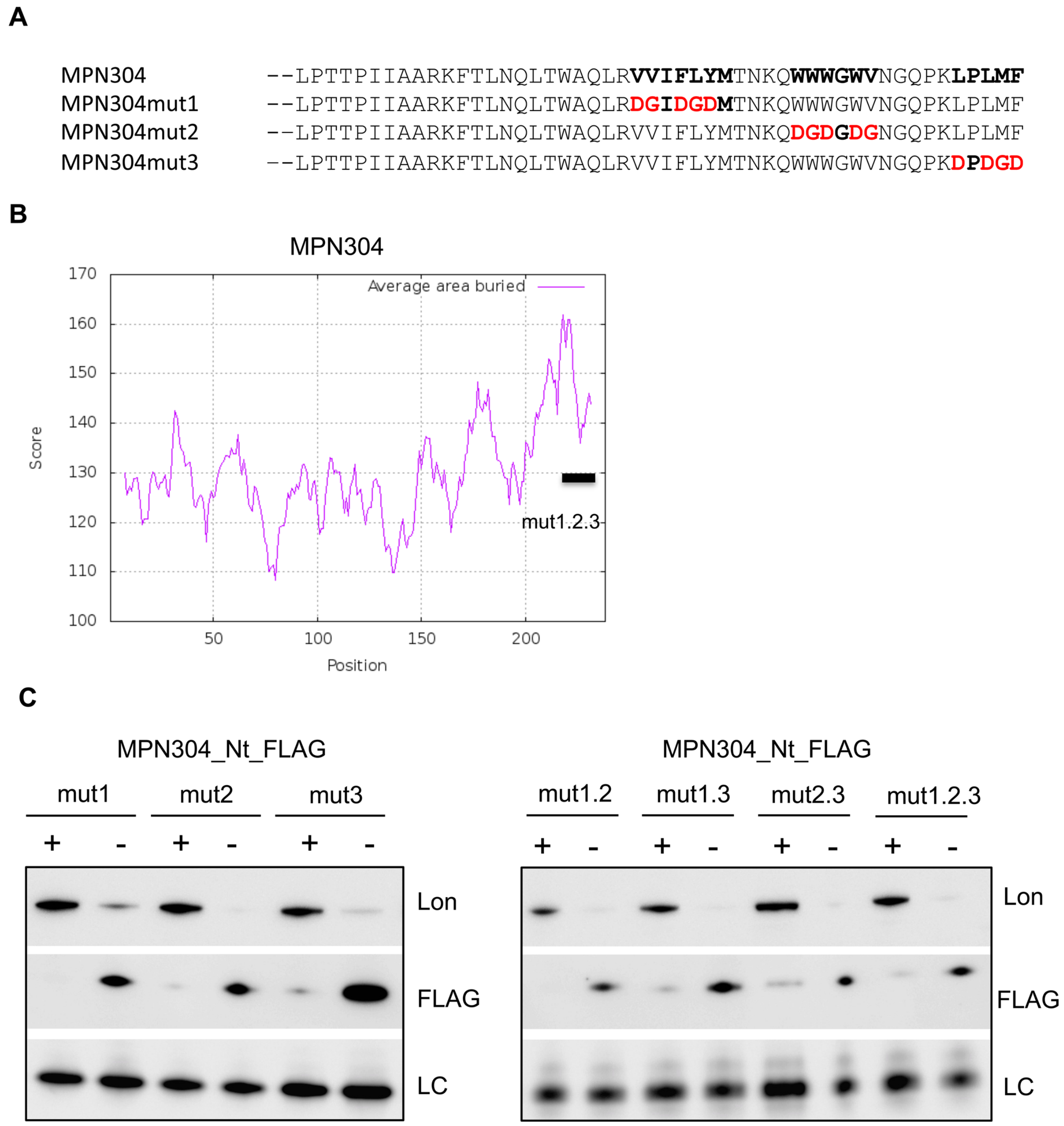

Appendix Figure S11. Mutational analysis to identify Lon degrons in MPN304 (ArcA Nt).

(A) Amino acid sequence of the last 50 residues of MPN304 and their mutant derivatives. Hydrophobic motifs that can potentially act as Lon degrons are highlighted in bold. Mutations performed in these putative Lon degrons are shown in red.

(B) Sequence analysis showing the surface-area burial score along MPN304 protein sequence. Analysis was performed using the ProtScale Tool selecting the "average area buried" amino acid scale (Gasteiger et al, 2005). Mutated regions in MPN304 are indicated in the plot.

(C) Protein stability assessment of MPN304 N-terminal FLAG-tagged derivatives containing single or combined mutations in putative Lon degrons. MPN304 mutant derivatives were expressed in the

ΔIndLon mutant and protein levels determined by immunoblot using anti-Lon and anti-FLAG antibodies comparing inducing (+) or depleting (–) conditions (48h of depletion). LC, loading control. We searched for sequence motifs in MPN304 with a high score for buried surface-area, as these regions have been shown to correlate well in *E. coli* with Lon-dependent degradation (Gur & Sauer, 2008). Although the C-terminal region of MPN304 has the highest score for buried surface-area of the protein and contains several motifs enriched in hydrophobic residues, only mutations in LPLMF located at the protein C-terminal end had a modest contribution in the stability of the protein (Appendix Figure S11C). The combination of mutated motifs in the C-terminal region did not further improve protein stability (Appendix Figure S11D), suggesting the existence of multiple exposed degrons.

Gasteiger E., Hoogland C., Gattiker A., Duvaud S., Wilkins M.R., Appel R.D., Bairoch A.; *Protein Identification and Analysis Tools on the ExPASy Server;* (In) John M. Walker (ed): The Proteomics Protocols Handbook, Humana Press (2005). pp. 571-607

Gur E & Sauer RT (2008) Recognition of misfolded proteins by Lon, a AAA protease. *Genes & Development* **22:** 2267–2277 Available at: http://dx.doi.org/10.1101/gad.1670908

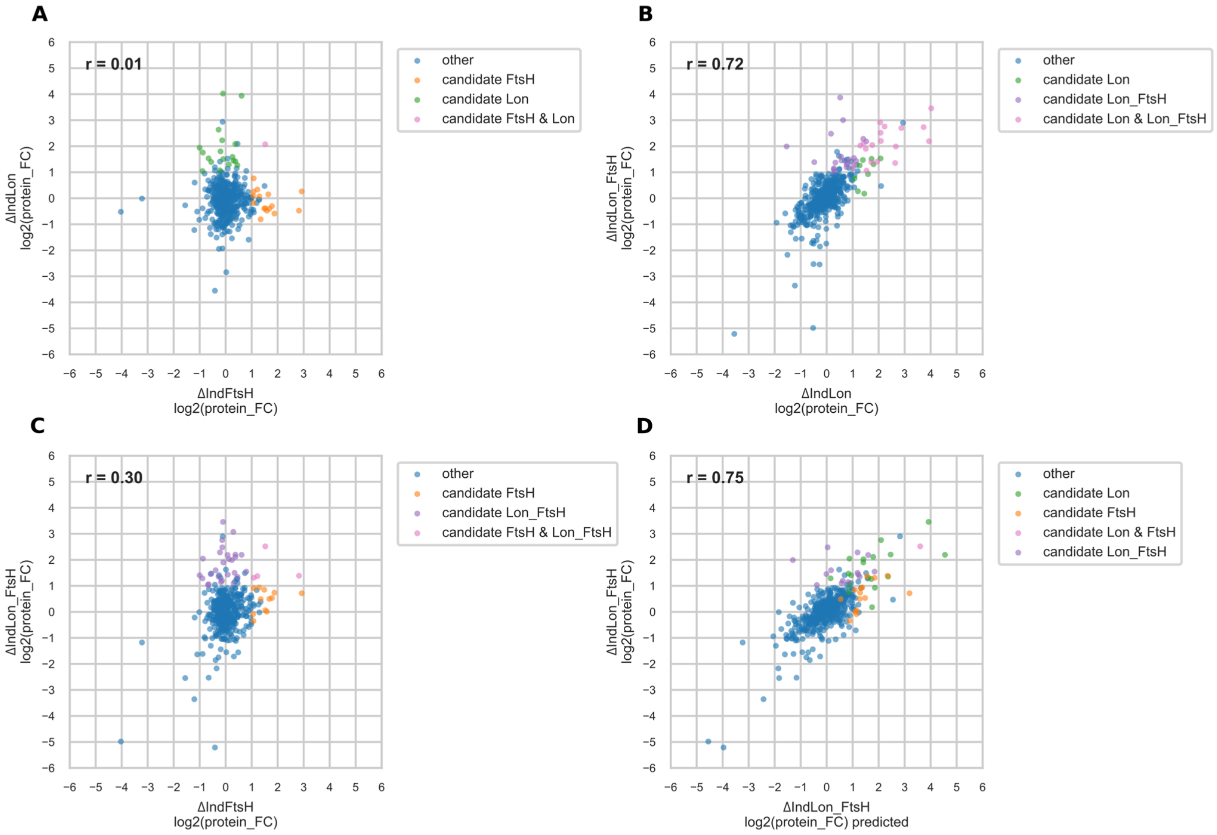

Appendix Figure S12. Correlation between proteome changes in responses to Lon and FtsH depletions. Correlation analysis of protein level changes comparing different mutant backgrounds. Data is reported as log_2 of protein fold changes observed after Lon and/or FtsH depletion (48h and 72h of depletion, respectively). **(A)** ΔIndLon v.s. ΔIndFtsH, **(B)** ΔIndLon v.s. ΔIndLon_FtsH, **(C)** ΔIndFtsH v.s. ΔIndLon_FtsH, **(D)** ΔIndLon_FtsH_predicted v.s. ΔIndLon_FtsH. The predicted changes in the double mutant (referred to as ΔIndLon_FtsH_predicted) were computed as the sum of the log_2 (protein_FC) in the two individual mutant experiments. Insets indicate pearson correlation coefficients.

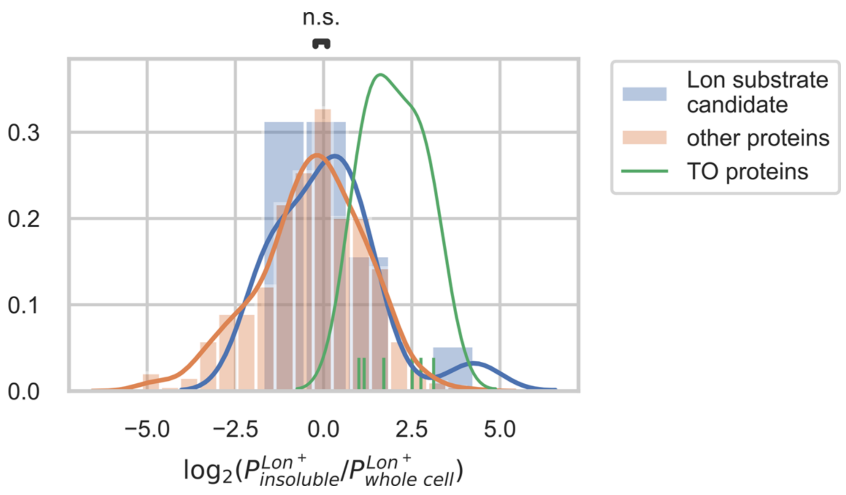

Appendix Figure S13. MS analysis showing the distribution of $\log_2$ fold-changes of the Triton X-100 insoluble fraction compared to the whole cell lysate, under Lon inducing conditions. Proteins associated with the terminal organelle (TO proteins), known to be enriched in the insoluble fractions, are shown as reference. No significant difference in $\log_2$ fold-changes for Lon substrate candidates was observed compared to the other proteins (Mann-Whitney-Wilcoxon two-sided test, p=0.08).

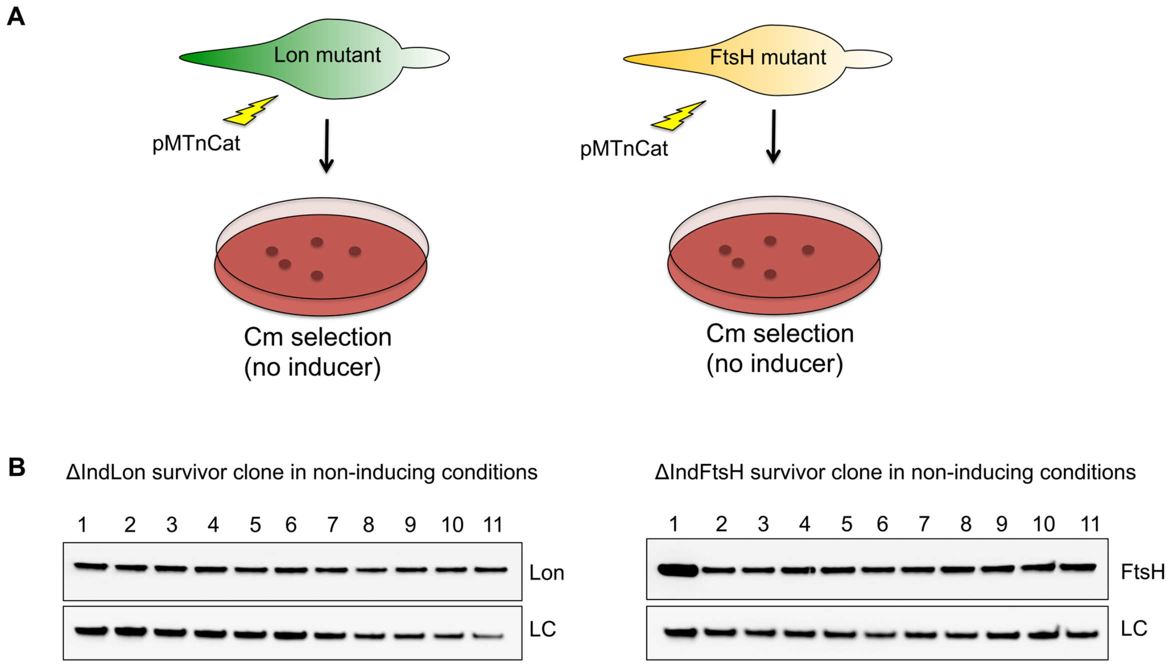

Appendix Figure S14. Transposon mutagenesis screening to identify putative gene mutations suppressing lethality associated with Lon and FtsH depletion.

(A). Schematic representation showing the screening strategy. Lon and FtsH conditional mutants in which the *cat* resistance gene was excised by the Cre-lox system were electroporated with the pMTnCat minitransposon. The resulting transformants were plated on Hayflick agar plates and selected in the absence of inducer and 20 µg/ml Cm. In these selecting conditions, only transformants having transposon insertions in genes that could cause the lethality of Lon or FtsH depletion, or with mutations that derepress the inducible system are expected in principle to be selected. Of note, this screening strategy has the limitation that the effect of essential genes cannot be evaluated. A total of 11 and approximately 100 colonies were obtained after two independent transformation experiments for the Lon and FtsH conditional mutants, respectively. **(B)** Western blot analysis assessing the expression of Lon and FtsH in 11 survivor clones from each conditional mutant revealed that all clones were capable to express Lon and FtsH, suggesting the presence of mutations affecting the repressing capabilities of the inducible system. LC, loading control.

Appendix Table S1. Mycoplasma strains used in this study.

Strain	Resistance	Reference
M129_GP35	$Puro^R$	Piñero-Lambea *et al*, 2020
ΔIndLon	$Puro^R$, Cm^R	This study
ΔIndLon_cat-	$Puro^R$, Cm^R	This study
M129_GP35_ pMTnTc_ftsH_Ind	$Puro^R$, Tc^R	This study
ΔIndFtsH	$Puro^R$, Cm^R, Tc^R	This study
ΔIndFtsH_cat-	$Puro^R$, Tc^R	This study
ΔIndLon_FtsH	$Puro^R$, Cm^R, Tc^R	This study
ΔIndLon_Δhmw2Nt	$Puro^R$, Cm^R	This study
ΔIndFtsH_pMTnCat_FLAG_MPN152	$Puro^R$, Cm^R, Tc^R	This study
ΔIndFtsH_pMTnCat_MPN152_FLAG	$Puro^R$, Cm^R, Tc^R	This study
ΔIndFtsH_pMTnCat_FLAG_MPN207a	$Puro^R$, Cm^R, Tc^R	This study
ΔIndFtsH_pMTnCat_MPN207a_FLAG	$Puro^R$, Cm^R, Tc^R	This study
ΔIndFtsH_pMTnCat_FLAG_MPN224	$Puro^R$, Cm^R, Tc^R	This study
ΔIndFtsH_pMTnCat_MPN224_FLAG	$Puro^R$, Cm^R, Tc^R	This study
ΔIndFtsH_pMTnCat_FLAG_MPN308	$Puro^R$, Cm^R, Tc^R	This study
ΔIndFtsH_pMTnCat_MPN308_FLAG	$Puro^R$, Cm^R, Tc^R	This study
ΔIndFtsH_pMTnCat_FLAG_MPN449	$Puro^R$, Cm^R, Tc^R	This study
ΔIndFtsH_pMTnCat_MPN449_FLAG	$Puro^R$, Cm^R, Tc^R	This study
ΔIndFtsH_pMTnCat_FLAG_MPN527	$Puro^R$, Cm^R, Tc^R	This study
ΔIndFtsH_pMTnCat_MPN527_FLAG	$Puro^R$, Cm^R, Tc^R	This study
ΔIndLon_pMTnTc_FLAG_MPN201	$Puro^R$, Cm^R, Tc^R	This study
ΔIndLon_pMTnTc_MPN201_FLAG	$Puro^R$, Cm^R, Tc^R	This study
ΔIndLon_pMTnTc_FLAG_MPN304	$Puro^R$, Cm^R, Tc^R	This study
ΔIndLon_pMTnTc_MPN304_FLAG	$Puro^R$, Cm^R, Tc^R	This study
ΔIndLon_pMTnTc_FLAG_MPN316	$Puro^R$, Cm^R, Tc^R	This study
ΔIndLon_pMTnTc_MPN316_FLAG	$Puro^R$, Cm^R, Tc^R	This study
ΔIndLon_pMTnTc_FLAG_MPN317	$Puro^R$, Cm^R, Tc^R	This study
ΔIndLon_pMTnTc_MPN317_FLAG	$Puro^R$, Cm^R, Tc^R	This study
ΔIndLon_pMTnTc_FLAG_MPN525	$Puro^R$, Cm^R, Tc^R	This study
ΔIndLon_pMTnTc_MPN525_FLAG	$Puro^R$, Cm^R, Tc^R	This study
ΔIndLon_pMTnTc_FLAG_MPN638	$Puro^R$, Cm^R, Tc^R	This study
ΔIndLon_pMTnTc_MPN638_FLAG	$Puro^R$, Cm^R, Tc^R	This study
ΔIndLon_pMTnTc_FLAG_MPN201mut1	$Puro^R$, Cm^R, Tc^R	This study
ΔIndLon_pMTnTc_MPN201_FLAGmut2	$Puro^R$, Cm^R, Tc^R	This study
ΔIndLon_pMTnTc_MPN201_FLAGmut3	$Puro^R$, Cm^R, Tc^R	This study
ΔIndLon_pMTnTc_MPN201_FLAGmut4	$Puro^R$, Cm^R, Tc^R	This study
ΔIndLon_pMTnTc_FLAG_MPN304mut1	$Puro^R$, Cm^R, Tc^R	This study
ΔIndLon_pMTnTc_FLAG_MPN304mut2	$Puro^R$, Cm^R, Tc^R	This study
ΔIndLon_pMTnTc_FLAG_MPN304mut3	$Puro^R$, Cm^R, Tc^R	This study
ΔIndLon_pMTnTc_FLAG_MPN304mut1.2	$Puro^R$, Cm^R, Tc^R	This study
ΔIndLon_pMTnTc_FLAG_MPN304mut1.3	$Puro^R$, Cm^R, Tc^R	This study
ΔIndLon_pMTnTc_FLAG_MPN304mut2.3	$Puro^R$, Cm^R, Tc^R	This study
ΔIndLon_pMTnTc_FLAG_MPN304mut1.2.3	$Puro^R$, Cm^R, Tc^R	This study
ΔIndLon_pMTnTc_FLAG_MPN316mut1	$Puro^R$, Cm^R, Tc^R	This study
ΔIndLon_pMTnTc_FLAG_MPN316mut2	$Puro^R$, Cm^R, Tc^R	This study
ΔIndLon_pMTnTc_FLAG_MPN316_L413D	$Puro^R$, Cm^R, Tc^R	This study
ΔIndLon_pMTnTc_FLAG_MPN316_V414G	$Puro^R$, Cm^R, Tc^R	This study
ΔIndLon_pMTnTc_FLAG_MPN316_L417D	$Puro^R$, Cm^R, Tc^R	This study
ΔIndLon_pMTnTc_FLAG_MPN316_I418G	$Puro^R$, Cm^R, Tc^R	This study
ΔIndLon_pMTnTc_FLAG_MPN317mut1	$Puro^R$, Cm^R, Tc^R	This study
ΔIndLon_pMTnTc_FLAG_MPN317mut2	$Puro^R$, Cm^R, Tc^R	This study
ΔIndLon_pMTnTc_FLAG_MPN317_F378D	$Puro^R$, Cm^R, Tc^R	This study
ΔIndLon_pMTnTc_FLAG_MPN317_Y380G	$Puro^R$, Cm^R, Tc^R	This study
ΔIndLon_pMTnTc_FLAG_MPN525mut1	$Puro^R$, Cm^R, Tc^R	This study
ΔIndLon_pMTnTc_FLAG_MPN525_V391G	$Puro^R$, Cm^R, Tc^R	This study
ΔIndLon_pMTnTc_FLAG_MPN525_Y393D	$Puro^R$, Cm^R, Tc^R	This study
ΔIndLon_pMTnTc_FLAG_MPN525_F394G	$Puro^R$, Cm^R, Tc^R	This study
ΔIndLon_pMTnTc_FLAG_MPN638CtMPN201	$Puro^R$, Cm^R, Tc^R	This study
ΔIndLon_pMTnTc_luc2_F89E	$Puro^R$, Cm^R, Tc^R	This study

Piñero-Lambea C, Garcia-Ramallo E, Martinez S, Delgado J, Serrano L & Lluch-Senar M (2020) Genome Editing Based on Oligo Recombineering and Cas9-Mediated Counterselection. *ACS Synth. Biol.* **9:** 1693–1704.

Appendix_Table S2. Gibson cloning strategy for each plasmid constructed in this study.

Gibson component	Primers	Template	Gibosn assembly
pΔLonPr_Ind			
PCR1	p_LA_Lon_F / ter_LA_LonR	gDNA M129	x
PCR2	ter_lox66_F / tetR_lox71_R	pMTnCat(lox) [1]	x
PCR3	lox71_tetR_F / RALonIndPr_R	pMTnParGP35eiCas9_eNT2 [2]	x
PCR4	IndPr_RALon_F/ p_RALon_R	gDNA M129	x
Vector		pBSK *Eco*RV digested	x
pMTnTc_ftsH_Ind			
PCR1	ter625_TetR_F / ftsH_IndPr_R	pΔLonPr_Ind [3]	
PCR2	p_ter625 _F / ftsH_IndPr_R	PCR1	x
PCR3	IndPr_LAFtsH_R / p_ftsH_R	gDNA M129	x
Vector		pMTnTetM438 *Eco*RV digested [4]	x
pΔFtsH			
PCR1	p_LA_ftsH _F / P438_LA_ftsH_R	gDNA M129	x
PCR2	P438_lox66 _F / RA_ftsH_lox71_R	pMTnCat(lox) [1]	x
PCR3	lox71_RA_ftsH_F / p_RA_ftsH_R	gDNA M129	x
Vector		pBSK *Eco*RV digested	x
pΔNt-hmw2			
PCR1	p_LA_hmw2_F / LA_hmw2_R	gDNA M129	x
PCR2	LA_hmw2_lox71_F / RA_hmw2_lox66_R	pMTnCat(lox) [1]	x
PCR3	RA_hmw2_F / p_RA_hmw2_R	gDNA M129	x
Vector		pBSK *Eco*RV digested	x
pMTnTc_luc2_F89E			
PCR1	P438_luc2_F / luc2_F89E_R	pMTnCat_luc2Ind_K [5]	
PCR2	p_P438_F / luc2_F89E_R	PCR1	x
PCR3	luc2_F89E_F / p_luc2_R2	PCR1	x
Vector		pMTnTetM438 *Eco*RV digested [4]	x
N-terminal FLAG variants of FtsH substrates (XXX indicates the corresponding gene number)			
PCR1	FLAG_MPNXXX_F / p_MPNXXX_R	gDNA M129	
PCR2	p_P438FLAG _F / p_MPNXXX_R	PCR 1	x
Vector		pMTnCat *Eco*RV digested [6]	x
C-terminal FLAG variants of FtsH substrates (XXX indicates the corresponding gene number)			
PCR1	P438_MPNXXX_F / FLAG_MPNXXX_R	gDNA M129	
PCR2	p_P438_F / p_FLAG_Ct_R	PCR 1	x
Vector		pMTnCat *Eco*RV digested [6]	x
N-terminal FLAG variants of Lon substrates (XXX indicates the corresponding gene number)			
PCR1	FLAG_MPNXXX_F / p_MPNXXX_R	gDNA M129	
PCR2	p_P438FLAG _F / p_MPNXXX_R	PCR 1	x
Vector		pMTnTetM438 *Eco*RV digested [4]	x
C-terminal FLAG variants of Lon substrates (XXX indicates the corresponding gene number)			
PCR1	P438_MPNXXX_F / FLAG_MPNXXX_R	gDNA M129	
PCR2	p_P438_F / p_FLAG_Ct_R	PCR 1	x
Vector		pMTnTetM438 *Eco*RV digested [4]	x
N and C- terminal FLAG variants with mutated degrons			
pMTnTc_FLAG_MPN201mut1			
PCR1	FLAG_MPN201 _F / MPN201_Ct638_R	*pMTnTc_FLAG_MPN201* [3]	
PCR2	p_P438FLAG _F / p_MPN638 _R	PCR 1	x
Vector		pMTnTetM438 *Eco*RV digested [4]	x
pMTnTc_MPN201_FLAGmut2			
PCR1	MPN201mut2_F / p_FLAG_Ct _R	*pMTnTc_MPN201_FLAG* [3]	
PCR2	P438_MPN201mut2_F/ p_FLAG_Ct _R	*PCR1*	
PCR3	p_P438_F / p_FLAG_Ct_R	PCR2	x
Vector		pMTnTetM438 *Eco*RV digested [4]	x

Appendix_Table S2. Gibson cloning strategy (cont.).

Gibson component	Primers	Template	Gibosn assembly
pMTnTc_MPN201_FLAGmut3			
PCR1	p_P438 _F / MPN201mut3_R	*pMTnTc_MPN201_FLAG* (3)	x
PCR2	MPN201mut3_F / p_FLAG_Ct _R	*pMTnTc_MPN201_FLAG* (3)	x
Vector		pMTnTetM438 *Eco*RV digested (4)	x
pMTnTc_MPN201_FLAGmut4			
PCR1	p_P438 _F / MPN201mut4_R	*pMTnTc_MPN201_FLAG* (3)	x
PCR2	MPN201mut4_F / p_FLAG_Ct _R	*pMTnTc_MPN201_FLAG* (3)	x
Vector		pMTnTetM438 *Eco*RV digested (4)	x
pMTnTc_FLAG_MPN304mut1			
PCR1	p_P438FLAG _F / MPN304mut1_R	*pMTnTc_FLAG_MPN304* (3)	x
PCR2	MPN304mut1_F / p_MPN304 _R	*pMTnTc_FLAG_MPN304* (3)	x
Vector		pMTnTetM438 *Eco*RV digested (4)	x
pMTnTc_FLAG_MPN304mut2			
PCR1	FLAG_MPN304 _F / MPN304mut2_R	*pMTnTc_FLAG_MPN304* (3)	
PCR2	p_P438FLAG _F / p_MPN304 _R	PCR1	x
Vector		pMTnTetM438 *Eco*RV digested (4)	x
pMTnTc_FLAG_MPN304mut3			
PCR1	p_P438FLAG _F / p_MPN304mut3 _R	*pMTnTc_FLAG_MPN304* (3)	x
Vector		pMTnTetM438 *Eco*RV digested (4)	x
pMTnTc_FLAG_MPN304mut1.2			
PCR1	p_P438FLAG _F / MPN304mut1_R	pMTnTc_FLAG_MPN304mut2(3)	x
PCR2	MPN304mut1_F/ p_MPN304 _R	pMTnTc_FLAG_MPN304mut2(3)	x
Vector		pMTnTetM438 *Eco*RV digested (4)	x
pMTnTc_FLAG_MPN304mut1.3			
PCR1	p_P438FLAG _F / MPN304mut1_R	*pMTnTc_FLAG_MPN304* (3)	x
PCR2	MPN304mut1_F/ p_MPN304mut3 _R	*pMTnTc_FLAG_MPN304* (3)	x
Vector		pMTnTetM438 *Eco*RV digested (4)	x
pMTnTc_FLAG_MPN304mut2.3			
PCR1	p_P438FLAG _F / p_MPN304mut3 _R	pMTnTc_FLAG_MPN304mut2(3)	x
Vector		pMTnTetM438 *Eco*RV digested (4)	x
pMTnTc_FLAG_MPN304mut1.2.3			
PCR1	p_P438FLAG _F / MPN304mut1_R	pMTnTc_FLAG_MPN304mut2(3)	x
PCR2	MPN304mut1_F/ p_MPN304mut3 _R	pMTnTc_FLAG_MPN304mut2(3)	x
Vector		pMTnTetM438 *Eco*RV digested (4)	x
pMTnTc_FLAG_MPN316mut1			
PCR1	FLAG_MPN316_F / MPN316mut1_R	*pMTnTc_FLAG_MPN316* (3)	
PCR2	p_P438FLAG _F / p_MPN316 _R	PCR1	x
Vector		pMTnTetM438 *Eco*RV digested (4)	x
pMTnTc_FLAG_MPN316mut2			
PCR1	FLAG_MPN316_F / MPN316mut2_R	*pMTnTc_FLAG_MPN316* (3)	
PCR2	p_P438FLAG _F / p_MPN316mut2 _R	PCR1	x
Vector		pMTnTetM438 *Eco*RV digested (4)	x
pMTnTc_FLAG_MPN316_L413D			
PCR1	p_P438FLAG *F* / p_MPN316_L413D_R	*pMTnTc_FLAG_MPN316* (3)	x
Vector		pMTnTetM438 *Eco*RV digested (4)	x
pMTnTc_FLAG_MPN316_V414G			
PCR1	p_P438FLAG *F* / p_MPN316_V414G_R	*pMTnTc_FLAG_MPN316* (3)	x
Vector		pMTnTetM438 *Eco*RV digested (4)	x
pMTnTc_FLAG_MPN316_L417D			
PCR1	p_P438FLAG *F* / p_MPN316_L417D_R	*pMTnTc_FLAG_MPN316* (3)	x
Vector		pMTnTetM438 *Eco*RV digested (4)	x

Appendix_Table S2. Gibson cloning strategy (cont.).

**Gibson component**	**Primers**	**Template**	**Gibosn assembly**
N and C- terminal FLAG variants with mutated degrons			
pMTnTc_FLAG_MPN316_I418G			
PCR1	p_P438FLAG *F* / p_MPN316_I418G_R	*pMTnTc_FLAG_MPN316* [3]	x
Vector		pMTnTetM438 *Eco*RV digested [4]	x
pMTnTc_FLAG_MPN317mut1			
PCR1	FLAG_MPN317 _F / MPN317mut1_R	*pMTnTc_FLAG_MPN317* [3]	
PCR2	p_P438FLAG _F / p_MPN317 _R	PCR1	x
Vector		pMTnTetM438 *Eco*RV digested [4]	x
pMTnTc_FLAG_MPN317mut2			
PCR1	FLAG_MPN317 _F / p_MPN317mut2_R	*pMTnTc_FLAG_MPN317* [3]	
PCR2	p_P438FLAG _F / p_MPN317mut2_R	PCR1	x
Vector		pMTnTetM438 *Eco*RV digested [4]	x
pMTnTc_FLAG_MPN317_F378D			
PCR1	p_P438FLAG *F* / p_MPN317_F378D_R	*pMTnTc_FLAG_MPN317* [3]	x
Vector		pMTnTetM438 *Eco*RV digested [4]	x
pMTnTc_FLAG_MPN317_Y380G			
PCR1	p_P438FLAG *F* / p_MPN317_Y380_R	*pMTnTc_FLAG_MPN317* [3]	x
Vector		pMTnTetM438 *Eco*RV digested [4]	x
pMTnTc_FLAG_MPN525mut1			
PCR1	FLAG_MPN525 _F / MPN525mut1_R	*pMTnTc_FLAG_MPN525* [3]	
PCR2	p_P438FLAG _F / MPN525mut1_R	PCR1	x
PCR3	MPN525mut1_F / p_MPN525 _R	*pMTnTc_FLAG_MPN525* [3]	x
Vector		pMTnTetM438 *Eco*RV digested [4]	x
pMTnTc_FLAG_MPN525_V391G			
PCR1	FLAG_MPN525 _F / MPN525_V391G_R	*pMTnTc_FLAG_MPN525* [3]	
PCR2	p_P438FLAG *F* / MPN525_V391G_R	PCR1	x
PCR3	MPN525_V391G_F / p_MPN525 _R	*pMTnTc_FLAG_MPN525* [3]	x
Vector		pMTnTetM438 *Eco*RV digested [4]	x
pMTnTc_FLAG_MPN525_Y393D			
PCR1	FLAG_MPN525 _F / MPN525_Y393D_R	*pMTnTc_FLAG_MPN525* [3]	
PCR2	p_P438FLAG *F* / MPN525_Y393D_R	PCR1	x
PCR3	MPN525_Y393D_F / p_MPN525 _R	*pMTnTc_FLAG_MPN525* [3]	x
Vector		pMTnTetM438 *Eco*RV digested [4]	x
pMTnTc_FLAG_MPN525_F394G			
PCR1	FLAG_MPN525 _F / MPN525_F394G_R	*pMTnTc_FLAG_MPN525* [3]	
PCR2	p_P438FLAG *F* / MPN525_F394G_R	PCR1	x
PCR3	MPN525_ F394G_F / p_MPN525 _R	*pMTnTc_FLAG_MPN525* [3]	x
Vector		pMTnTetM438 *Eco*RV digested [4]	x
pMTnTc_FLAG_MPN638CtMPN201			
PCR1	FLAG_MPN638 _F / MPN638_Ct201_R	*pMTnTc_FLAG_MPN638* [3]	
PCR2	p_P438FLAG _F / p_MPN201 _R	PCR1	x
Vector		pMTnTetM438 *Eco*RV digested [4]	x

(1) In-house cloning vector containing the *cat* gene flanked by lox sites.

(2) Piñero-Lambea C, Garcia-Ramallo E, Martinez S, Delgado J, Serrano L & Lluch-Senar M (2020) Genome Editing Based on Oligo Recombineering and Cas9-Mediated Counterselection. *ACS Synth. Biol.* **9:** 1693–1704.

(3) This study.

[4] Pich, O.Q., Burgos, R., Planell, R., Querol, E., and Piñol, J. (2006). Comparative analysis of antibiotic resistance gene markers in Mycoplasma genitalium: application to studies of the minimal gene complement. Microbiology 152, 519–527.
[5] Weber M, Burgos R, Yus E, Yang J-S, Lluch-Senar M & Serrano L (2020) Impact of C-terminal amino acid composition on protein expression in bacteria. *Mol. Syst. Biol.* **16:** e9208
[6] Burgos, R., Wood, G.E., Young, L., Glass, J.I., and Totten, P.A. (2012). RecA mediates MgpB and MgpC phase and antigenic variation in Mycoplasma genitalium, but plays a minor role in DNA repair. Mol. Microbiol. 85, 669–683.

Appendix_Table S3. Primers used in this study.

Primer name	Sequence (5' to 3')[a]
Construction of plasmids	
**pΔLonPr_Ind**	
p_LALon _F	ACGGTATCGATAAGCTTGATACTTACTTTCATAAGTTAGTTTC
ter_LALon _R	ATACCTGAAAGACTCAGGTATTTTTTGAGTGCTAATGAGTGGAGTA
ter_lox66 _F	TACCTGAGTCTTTCAGGTATTTTTTTACCGTTCGTATAGCATAC
tetR _lox71_R	TGGGTCTTAATACCGTTCGTATAATGTATG
lox71_tetR _F	ACGAACGGTATTAAGACCCACTTTCACATTTA
RALon_IndPr_R	CAGCTGGCATATGCTCTATCAATGATAGAGGA
IndPr_RALon_F	GATAGAGCATATGCCAGCTGTAAAAAAACCA
p_RALon_R	CCGGGCTGCAGGAATTCGATTCTTTTGGTACCTTAGATAGC
**pMTnTc_ftsH_Ind**	
p_ter625 _F	ACGGTATCGATAAGCTTGATAAAAAATACCTGAGTCTTTCAGGTATT
ter625_TetR_F	TACCTGAGTCTTTCAGGTATTTTTTTTAAGACCCACTTTCACATTTA
ftsH_IndPr_R	TTTTTTCATATGCTCTATCAATGATAGAGG
IndPr_LAFtsH_R	GATAGAGCATATGAAAAAAAATAAAGGACTTAACG
p_ftsH_R	TCTAAATACTAGAATTCGATTTAACTGTTTGTTTCACTGTCT
**pΔFtsH**	
p_LA_ftsH _F	ACGGTATCGATAAGCTTGATCTTGTACTGCCTTTTGGTGC
P438_LA_ftsH_R	GTATTTAGAATTAATAAAGTGCAAGAACAGCAAGCCAAAC
P438_lox66 _F	ACTTTATTAATTCTAAATACTATACCGTTCGTATAGCATAC
RA_ftsH_lox71_R	ACTACGAGCGAAAAACCGCATACCGTTCGTATAATGTATG
lox71_RA_ftsH_F	TGCGGTTTTTCGCTCGTAGT
p_RA_ftsH_R	CCGGGCTGCAGGAATTCGATCCTAAAGGCAAGGACATTCTT
**pΔNt-hmw2**	
p_LA_hmw2_F	ACGGTATCGATAAGCTTGATCGCCATCTTTTCGATGCGC
LA_hmw2_R	CGGTTAAAAGAGCCAGTGTT
LA_hmw2_lox71_F	AACACTGGCTCTTTTAACCGTACCGTTCGTATAATGTATG
RA_hmw2_lox66_R	CTGTTCTAAGTGTTTAGCAATACCGTTCGTATAGCATAC
RA_hmw2_F	TTGCTAAACACTTAGAACAGCA
p_RA_hmw2_R	CCGGGCTGCAGGAATTCGATGTCAGCTTGGTACTGCCTT
**pMTnTc_luc2_F89E**	
p_P438 _F	ACGGTATCGATAAGCTTGATTAGTATTTAGAATTAATAAAGTATG
luc2_F89E_R	ACACGGGCAT*TTC*GAACTGCAAG
P438_luc2_F	TAGTATTTAGAATTAATAAAGTATGGAAGATGCCAAAAACATTA
luc2_F89E_F	CTTGCAGTTC*GAA*ATGCCCGTGT
p_luc2_R2	TCTAAATACTAGAATTCGATTTACACGGCGATCTTGCCG
Production of ssDNA recombineering substrates and PCR screening	
Bio_lonPr_F	[biotin]ACTTACTTTCATAAGTTAGTTTC
Pro_lonPr_R	T*C*T*T*TTGGTACCTTAGATAGC
Pro_KOftsH_F	C*T*T*G*TACTGCCTTTTGGTGC
Bio_KOftsH_R	[biotin]CCTAAAGGCAAGGACATTCTT
Pro_KOhmw2_R	G*T*C*A*GCTTGGTACTGCCTT
Bio_KOhmw2_F	[biotin]CGCCATCTTTTCGATGCGC
Construction of N- and C-terminal FLAG variants	
p_P438_F	ACGGTATCGATAAGCTTGATTAGTATTTAGAATTAATAAAGTATG
p_FLAG_Ct_R	TCTAAATACTAGAATTCGATTTATTTGTCATCATCGTCCTTGTAGTC
p_P438FLAG _F	ACGGTATCGATAAGCTTGATTAGTATTTAGAATTAATAAAGTATGGACTACAAGG
FLAG_MPN152_F	AATAAAGTATGGACTACAAGGACGATGATGACAAAAATTTAAGTACGGCGCTATT
p_MPN152_R	TCTAAATACTAGAATTCGATCTATTCCATCACGGAAACGA
P438_MPN152_F	TAGTATTTAGAATTAATAAAGTATGAAATTTAAGTACGGCGCT
FLAG_MPN152_R	TCATCATCGTCCTTGTAGTCTTCCATCACGGAAACGACC
FLAG_MPN207a_F	AATAAAGTATGGACTACAAGGACGATGATGACAAACGAAAAGGTTGTATACGCATG
p_MPN207a_R	TCTAAATACTAGAATTCGATTTACTTAAACATTGAGGGAATG
P438_MPN207a_F	TAGTATTTAGAATTAATAAAGTATGCGAAAAGGTTGTATACGC
FLAG_MPN207a_R	TCATCATCGTCCTTGTAGTCCTTAAACATTGAGGGAATGGA
FLAG_MPN224_F	AATAAAGTATGGACTACAAGGACGATGATGACAAAAATCCTAGTGTTAGTAGCAG
p_MPN224_R	TCTAAATACTAGAATTCGATCTAAGCTTCCGGCACTGC
P438_MPN224_F	TAGTATTTAGAATTAATAAAGTATGAATCCTAGTGTTAGTAGC
FLAG_MPN224_R	TCATCATCGTCCTTGTAGTCAGCTTCCGGCACTGCTTC
FLAG_MPN308_F	AATAAAGTATGGACTACAAGGACGATGATGACAAAAAACAACAAAAACCCAAAATTAG
p_MPN308_R	TCTAAATACTAGAATTCGATTTAACCGTTAATGGTGGAGT
P438_MPN308_F	TAGTATTTAGAATTAATAAAGTATGAAACAACAAAAACCCAAA
FLAG_MPN308_R	TCATCATCGTCCTTGTAGTCACCGTTAATGGTGGAGTG
FLAG_MPN449_F	AATAAAGTATGGACTACAAGGACGATGATGACAAAACTTTTAGCGATCTTTTAACCA
p_MPN449_R	TCTAAATACTAGAATTCGATTTACCCAAAAACAGCTGGGG
P438_MPN449_F	TAGTATTTAGAATTAATAAAGTATGACTTTTAGCGATCTTTTAAC
FLAG_MPN449_R	TCATCATCGTCCTTGTAGTCCCCAAAAACAGCTGGGGGT
FLAG_MPN527_F	AATAAAGTATGGACTACAAGGACGATGATGACAAAAATGGTGCAAGAATAGCTTT
p_MPN527_R	TCTAAATACTAGAATTCGATTTAATAGACAATCTGCGCTTTC
P438_MPN527_F	TAGTATTTAGAATTAATAAAGTATGAATGGTGCAAGAATAGC
FLAG_MPN527_R	TCATCATCGTCCTTGTAGTCATAGACAATCTGCGCTTTCT

Appendix_Table S3. Primers used in this study (cont.).

Primer name	Sequence (5' to 3')[a]
Construction of N- and C-terminal FLAG variants (cont.)	
FLAG_MPN201_F	AATAAAGTATGGACTACAAGGACGATGATGACAAAGAGATTAAAACTTACAAAATCA
p_MPN201 _R	TCTAAATACTAGAATTCGATCTAATTTCTTATTTTTTGAACTCAATTAAATAAGAAG
P438_MPN201 _F	TAGTATTTAGAATTAATAAAGTATGGAGATTAAAACTTACAAAATC
FLAG_MPN201 _R	TCATCATCGTCCTTGTAGTCATTTCTTATTTTTTGAACTCAATT
FLAG_MPN304 _F	AATAAAGTATGGACTACAAGGACGATGATGACAAAAAGTACAACATCAACGTTCAT
p_MPN304 _R	TCTAAATACTAGAATTCGATCTAAAACATTAATGGCAGCTTC
P438_MPN304 _F	TAGTATTTAGAATTAATAAAGTATGAAGTACAACATCAACGTTC
FLAG_MPN304 _R	TCATCATCGTCCTTGTAGTCAAACATTAATGGCAGCTTCGG
FLAG_MPN316_F	AATAAAGTATGGACTACAAGGACGATGATGACAAATATAACCTCAAAAACATCTACG
p_MPN316 _R	TCTAAATACTAGAATTCGATTTACTTTATTAGTTTTTGCACTA
P438_MPN316 _F	TAGTATTTAGAATTAATAAAGTATGTATAACCTCAAAAACATCTAC
FLAG_MPN316_R	TCATCATCGTCCTTGTAGTCCTTTATTAGTTTTTGCACTAACT
FLAG_MPN317 _F	AATAAAGTATGGACTACAAGGACGATGATGACAAAGATTGAATACAAACAGCAGG
p_MPN317 _R	TCTAAATACTAGAATTCGATCTAATAATTAAATCCGGTTTGC
P438_MPN317_F	TAGTATTTAGAATTAATAAAGTATGGATTGAATACAAACAGCA
FLAG_MPN317_R	TCATCATCGTCCTTGTAGTCATAATTAAATCCGGTTTGCTG
FLAG_MPN525 _F	AATAAAGTATGGACTACAAGGACGATGATGACAAACAGCCGAACTATTACCGTG
p_MPN525 _R	TCTAAATACTAGAATTCGATTTAGCGTTTGTGTTTTCCATTT
P438_MPN525 _F	TAGTATTTAGAATTAATAAAGTATGCAGCCGAACTATTACC
FLAG_MPN525 _R	TCATCATCGTCCTTGTAGTCGCGTTTGTGTTTTCCATTTTCC
FLAG_MPN638 _F	AATAAAGTATGGACTACAAGGACGATGATGACAAAACTCCTAAATTAAAGCTTAACA
p_MPN638 _R	TCTAAATACTAGAATTCGATTTAAGATAGTGTTGGGAACAATTTGCCCAA
P438_MPN638 _F	TAGTATTTAGAATTAATAAAGTATGACTCCTAAATTAAAGCTTAA
FLAG_MPN638 _R	TCATCATCGTCCTTGTAGTCAGATAGTGTTGGGAACAATTTG
Construction of N- and C-terminal FLAG variants with mutated degrons	
MPN201_Ct638_R	TTGGGAACAATTTGCCCAATAAGTTATCGCGAATACTATCCAATTGCTTTTTACGCA
MPN201mut2_F	*GGC*AAAACT*GAC*AAA*GGC*AAAGAT*GGC*TGTGAT*GGC*ACACGCGGTAGAGTTATC
P438_MPN201mut2_F	TAGTATTTAGAATTAATAAAGTATGGAGGGCAAAACTGACAAAGGC
MPN201mut3_R	CGA*GCCAT*CAGG*GCCAT*CTCCTGGATCTTTTTTAATGTC
MPN201mut3_F	GAG*A*T*GGC*CCTG*A*T*GGC*TCGGCAGCTACTAATAACGA
MPN201mut4_R	TA*C*CAT*C*TTCACCATC*GCC*GTCA*CC*AGAATTTATTCGACCAAACT
MPN201mut4_F	C*GGC*GATGGTGAA*G*ATG*G*TACT*GAT*ACAGCTGACGGTTATGG
MPN304mut1_R	ATAT*CCCCATC*TAT*CCCGT*CCCTCAATTGTGCCCATGT
MPN304mut1_F	G*ACGGG*ATA*GA*T*GG*GGATATGACCAACAAACAG
MPN304mut2_R	AAACATTAATGGCAGCTTCGGTTGTCCGTTC*C*CGTCACC*GTC*TC*CGTC*CTGTTTG
p_MPN304mut3 _R	TCTAAATACTAGAATTCGATCTAA*TCCCCGTC*TGG*GTC*CTTCGGTTGTCCGTTC
MPN316mut1_R	CTTTATTAGTTTTTGCACTAACTTATTAATGTGCGTGTG*AT*CCTT*GCCATCGCCATC*CGACTTTTTAAACTGC
MPN316mut2_R	*GCCATC*TTTTTG*GCCATC*CTTATTAATGTGCGTGTGTAC
p_MPN316mut2 _R	TCTAAATACTAGAATTCGATTTACTT*GCCATC*TTTTTG*GCCATC*C
p_MPN316_L413D_R	TCTAAATACTAGAATTCGATTTACTTTATTAGTTTTTGCAC*ATC*CTTATTAATGT
p_MPN316_V414G _R	TCTAAATACTAGAATTCGATTTACTTTATTAGTTTTTG*GCC*TAACTTATTAATGT
p_MPN316_L417D_R	TCTAAATACTAGAATTCGATTTACTTTAT*ATC*TTTTTGCACTAACTTATTAATGT
p_MPN316_I418G_R	TCTAAATACTAGAATTCGATTTACTT*GCC*TAGTTTTTGCACTAACTTATTAATGT
MPN317mut1_R	CTAATAATTAAATCCGGTTTGCTGTTTG*C*C*A*TCTTT*GCC*TCC*ATC*CTCCTTTAAATC
p_MPN317mut2_R	TCTAAATACTAGAATTCGATCTA*GCC*ATTA*TC*TCCGGTTTGCTGTTT
p_MPN317_F378D_R	TCTAAATACTAGAATTCGATCTAATAATTA*TC*TCCGGTTTGCTGTTT
p_MPN317_Y380G_R	TCTAAATACTAGAATTCGATCTA*GCC*ATTAAATCCGGTTTGCTGTTT
MPN525mut1_F	CTGATGACG*G*CAAT*G*AC*GG*TTTAAACCGT
MPN525mut1_R	ACGGTTTAAA*CC*GT*C*ATTG*C*CGTCATCAG
MPN525_V391G_F	CTGATGACG*G*CAATTACTTTTTAAACCGT
MPN525_V391G_R	ACGGTTTAAAAAGTAATTG*C*CGTCATCAG
MPN525_Y393D_F	CTGATGACGTCAAT*G*ACTTTTTAAACCGT
MPN525_Y393D _R	ACGGTTTAAAAAGT*C*ATTGACGTCATCAG
MPN525_F394G_F	CTGATGACGTCAATTAC*GG*TTTAAACCGT
MPN525_F394G _R	ACGGTTTAAA*CC*GTAATTGACGTCATCAG
MPN638_Ct201_R	TTGAACTCAATTAAATAAGAAGTTCTGATAGTAGATTAAAGACTTAGCTTCTTC

[a] The underlined sequences indicate the priming sequences. Depending on the primer, the remaining sequence of the primer was designed to introduce promoter and terminator sequences, mutations (in italic bases) or overlapping sequences for Gibson assembly.
