# Supplementary material for: Protein quality control and regulated proteolysis in the genome‐reduced organism Mycoplasma pneumoniae
Source: Mol Syst Biol. 2020 Dec 15;16(12):e9530. doi: 10.15252/msb.20209530 (PMC7737663; doi:10.15252/msb.20209530)
Supplement: Supplementary file 2 — Expanded View Figures PDF [file MSB-16-e9530-s002.pdf]

Expanded View Figures

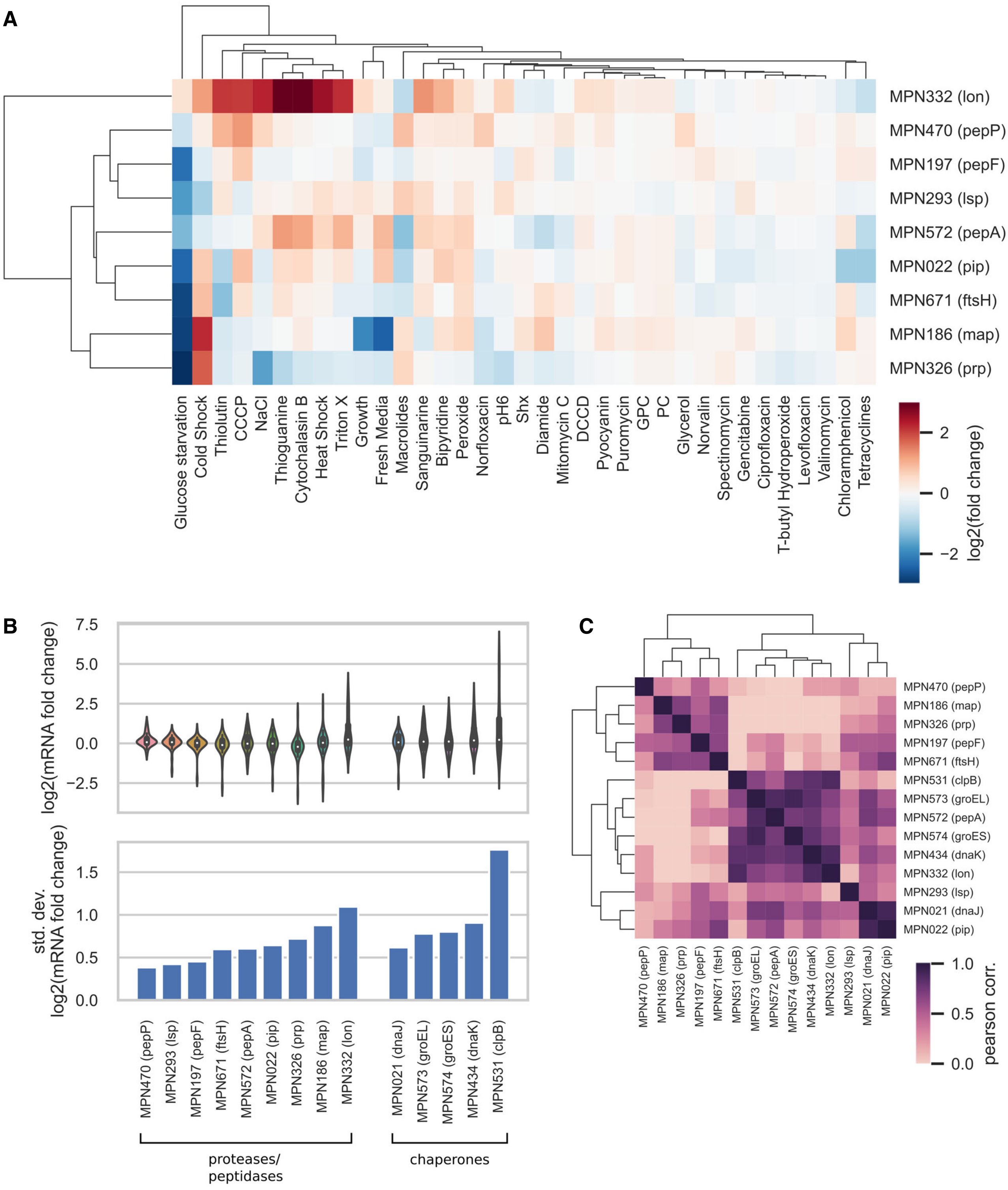

Figure EV1.

Figure EV1. Variability of transcriptional changes across perturbations for chaperons and proteases.

A–C Analysis of transcriptional changes of proteases, peptidases, and chaperones across a set of 35 environmental and genetic perturbations taken from Yus *et al* (2019). (A) Fold changes in mRNA levels for proteases and peptidases. Genes and perturbations were clustered based on the similarity of their transcriptional change pattern. (B) Distribution (upper plot) and standard deviation (lower plot) of the $\log_2$ of mRNA fold changes for proteases, peptidases, and chaperones. (C) Pearson correlation coefficient of mRNA fold changes between pairs of genes in the chaperones, proteases, and peptidases functional groups. Genes were clustered based on the similarity of their correlation pattern.

Figure EV2. Transcriptional responses to Lon and FtsH depletions.

A–D Correlation analysis of transcriptional changes comparing different mutant backgrounds. Data are reported as $\log_2$ of mRNA fold changes observed after Lon and/or FtsH depletion (48 and 72 h of depletion, respectively). (A) ΔIndLon vs. ΔIndFtsH, (B) ΔIndLon vs. ΔIndLon_FtsH, (C) ΔIndFtsH vs. ΔIndLon_FtsH, (D) ΔIndLon_FtsH_predicted vs. ΔIndLon_FtsH. The predicted changes in the double mutant (referred to as ΔIndLon_FtsH_predicted) were computed as the sum of the $\log_2$(mRNA_FC) in the two individual mutant experiments. Insets indicate Pearson correlation coefficients.

E–G Examples illustrating combination of regulatory changes in response to the simultaneous depletion of Lon and FtsH: (E) synergic effect when genes are transcriptionally upregulated ($\log_2$(mRNA_FC) > 1), or (F) downregulated in both individual mutants ($\log_2$(mRNA_FC) < −0.6). (G) Ribosomal operons containing genes *mpn164* to *mpn196*, showing opposite transcriptional responses in both individual mutants that are neutralized after the simultaneous depletion of both proteases.

© 2020 The Authors

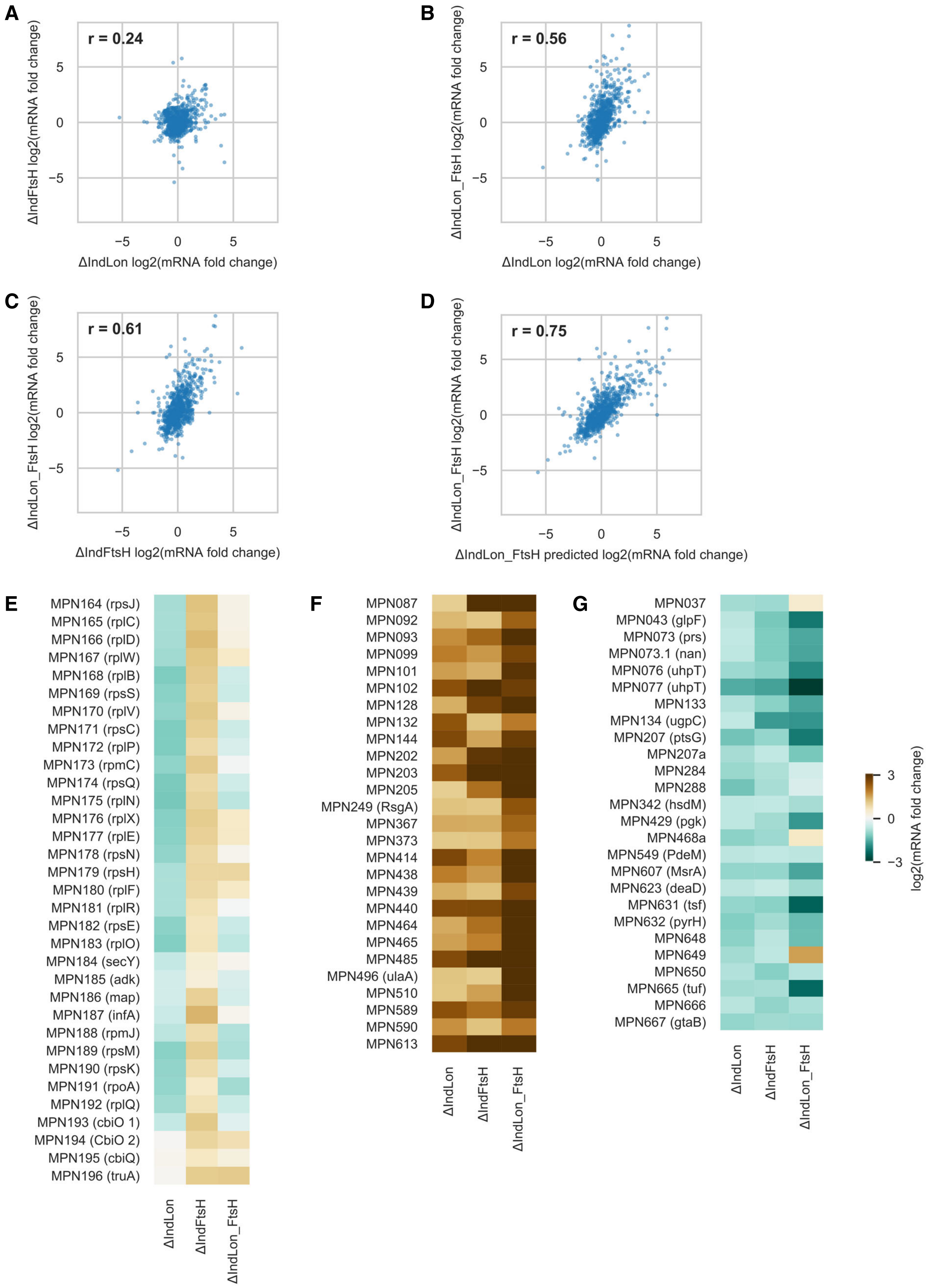
A
r = 0.24
ΔIndFtsH log2(mRNA fold change)
ΔIndLon log2(mRNA fold change)
B
r = 0.56
ΔIndLon_FtsH log2(mRNA fold change)
ΔIndLon log2(mRNA fold change)
C
r = 0.61
ΔIndLon_FtsH log2(mRNA fold change)
ΔIndFtsH log2(mRNA fold change)
D
r = 0.75
ΔIndLon_FtsH log2(mRNA fold change)
ΔIndLon_FtsH predicted log2(mRNA fold change)
E
MPN164 (rpsJ)
MPN165 (rplC)
MPN166 (rplD)
MPN167 (rplW)
MPN168 (rplB)
MPN169 (rpsS)
MPN170 (rplV)
MPN171 (rpsC)
MPN172 (rplP)
MPN173 (rpmC)
MPN174 (rpsQ)
MPN175 (rplN)
MPN176 (rplX)
MPN177 (rplE)
MPN178 (rpsN)
MPN179 (rpsH)
MPN180 (rplF)
MPN181 (rplR)
MPN182 (rpsE)
MPN183 (rplO)
MPN184 (secY)
MPN185 (adk)
MPN186 (map)
MPN187 (infA)
MPN188 (rpmJ)
MPN189 (rpsM)
MPN190 (rpsK)
MPN191 (rpoA)
MPN192 (rplQ)
MPN193 (cbiO 1)
MPN194 (CbiO 2)
MPN195 (cbiQ)
MPN196 (truA)
ΔIndLon
ΔIndFtsH
ΔIndLon_FtsH
F
MPN087
MPN092
MPN093
MPN099
MPN101
MPN102
MPN128
MPN132
MPN144
MPN202
MPN203
MPN205
MPN249 (RsgA)
MPN367
MPN373
MPN414
MPN438
MPN439
MPN440
MPN464
MPN465
MPN485
MPN496 (ulaA)
MPN510
MPN589
MPN590
MPN613
ΔIndLon
ΔIndFtsH
ΔIndLon_FtsH
G
MPN037
MPN043 (glpF)
MPN073 (prs)
MPN073.1 (nan)
MPN076 (uhpT)
MPN077 (uhpT)
MPN133
MPN134 (ugpC)
MPN207 (ptsG)
MPN207a
MPN284
MPN288
MPN342 (hsdM)
MPN429 (pgk)
MPN468a
MPN549 (PdeM)
MPN607 (MsrA)
MPN623 (deaD)
MPN631 (tsf)
MPN632 (pyrH)
MPN648
MPN649
MPN650
MPN665 (tuf)
MPN666
MPN667 (gtaB)
ΔIndLon
ΔIndFtsH
ΔIndLon_FtsH
log2(mRNA fold change)

Figure EV2.
