# Supplementary material for: Protein quality control and regulated proteolysis in the genome‐reduced organism Mycoplasma pneumoniae
Source: Mol Syst Biol. 2020 Dec 15;16(12):e9530. doi: 10.15252/msb.20209530 (PMC7737663; doi:10.15252/msb.20209530)

Appendix Figure S10

Fig. S10C (MPN201_Nt_FLAGmut1(CtMPN638))

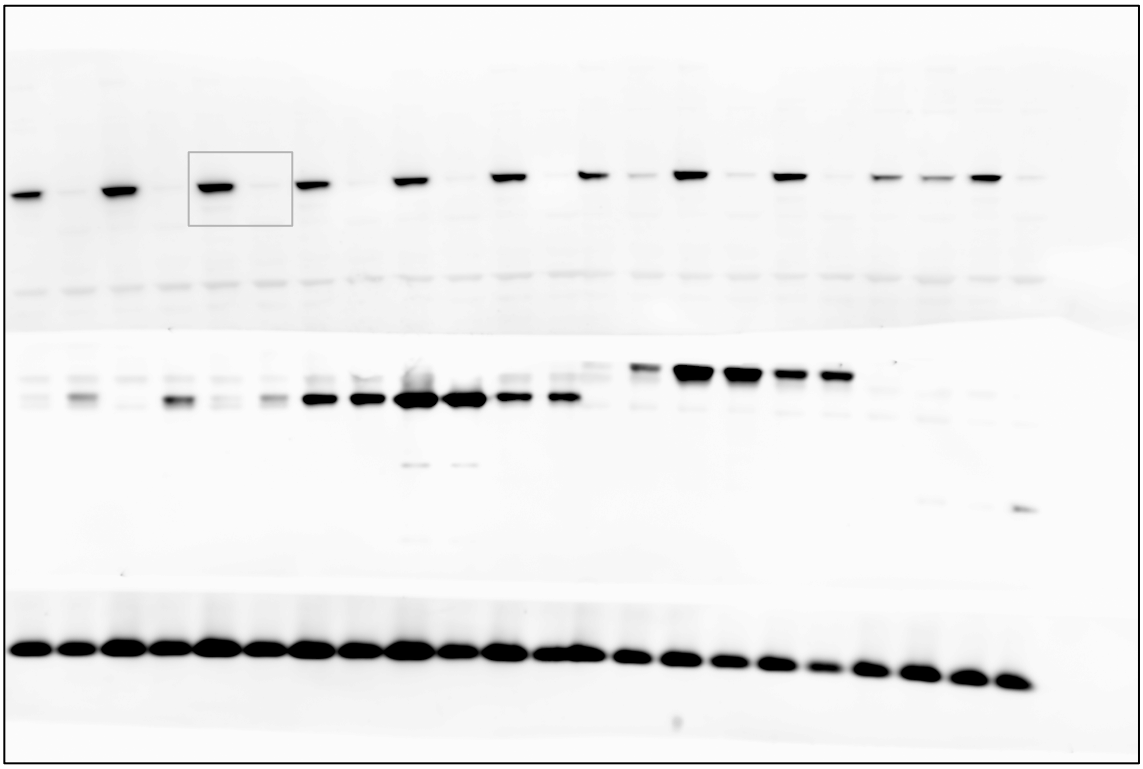

Lon

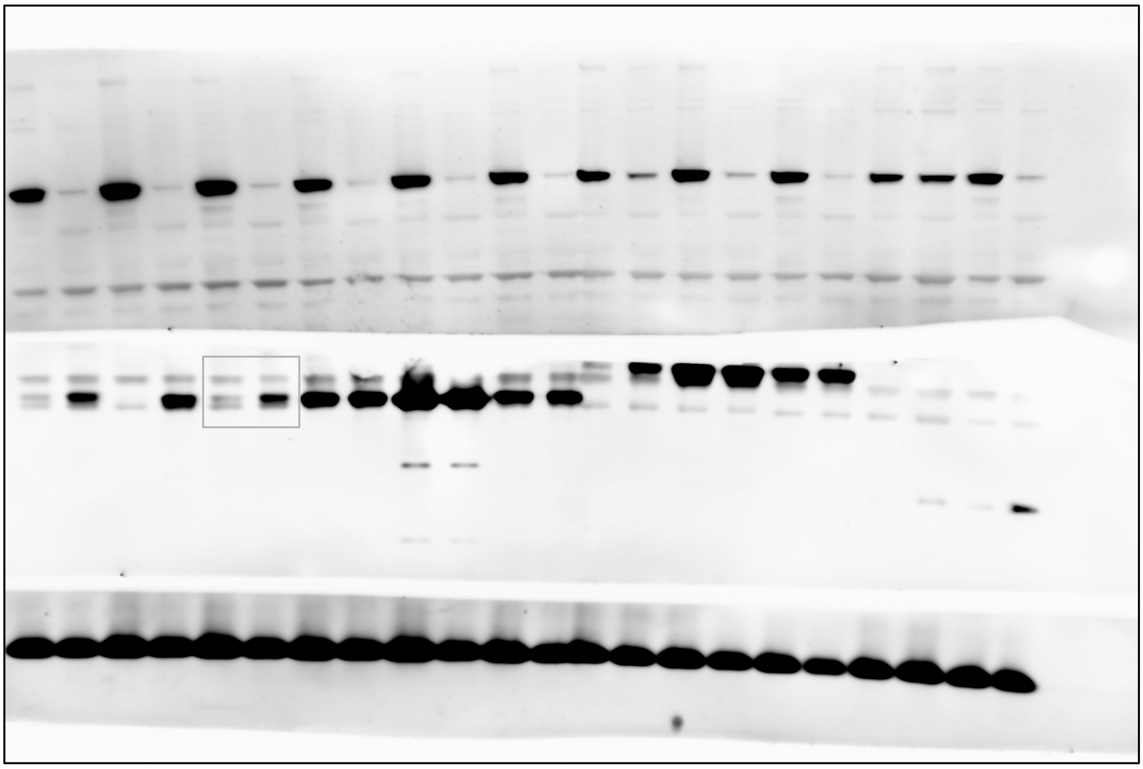

FLAG

Fig. S10C (MPN201_Nt_FLAGmut1(CtMPN638)). cont

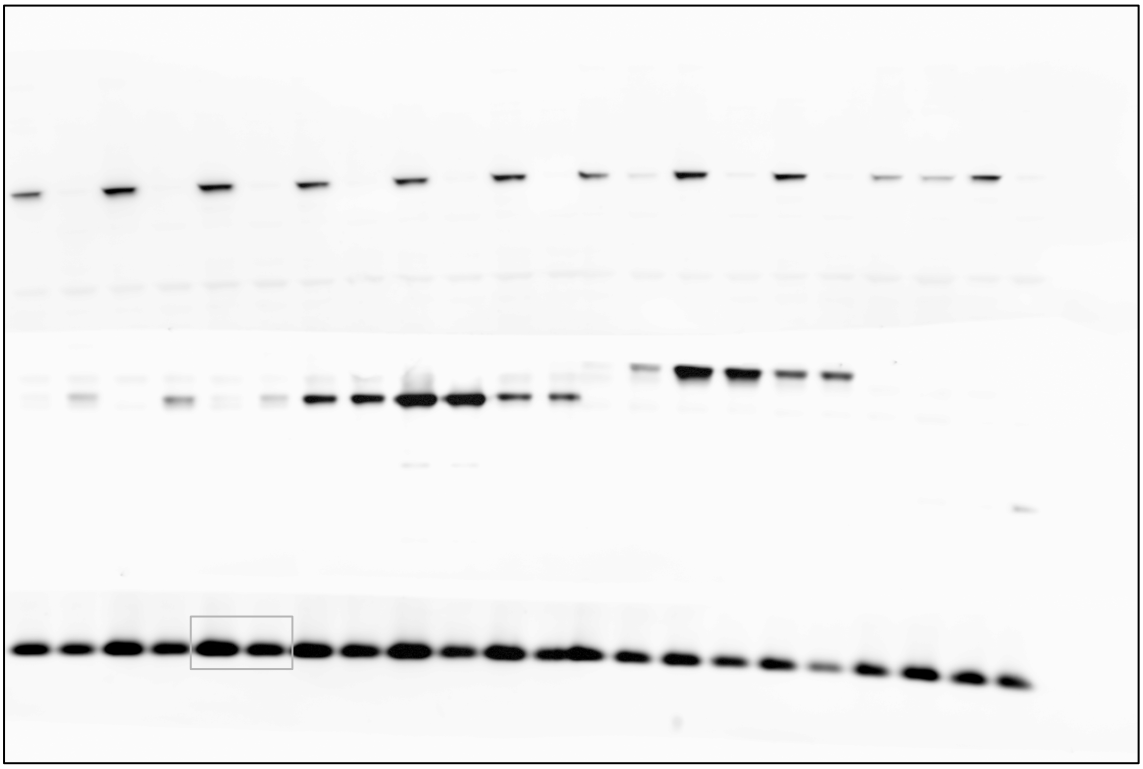

Fig. S10C (MPN638_Nt_FLAG (CtMPN201))

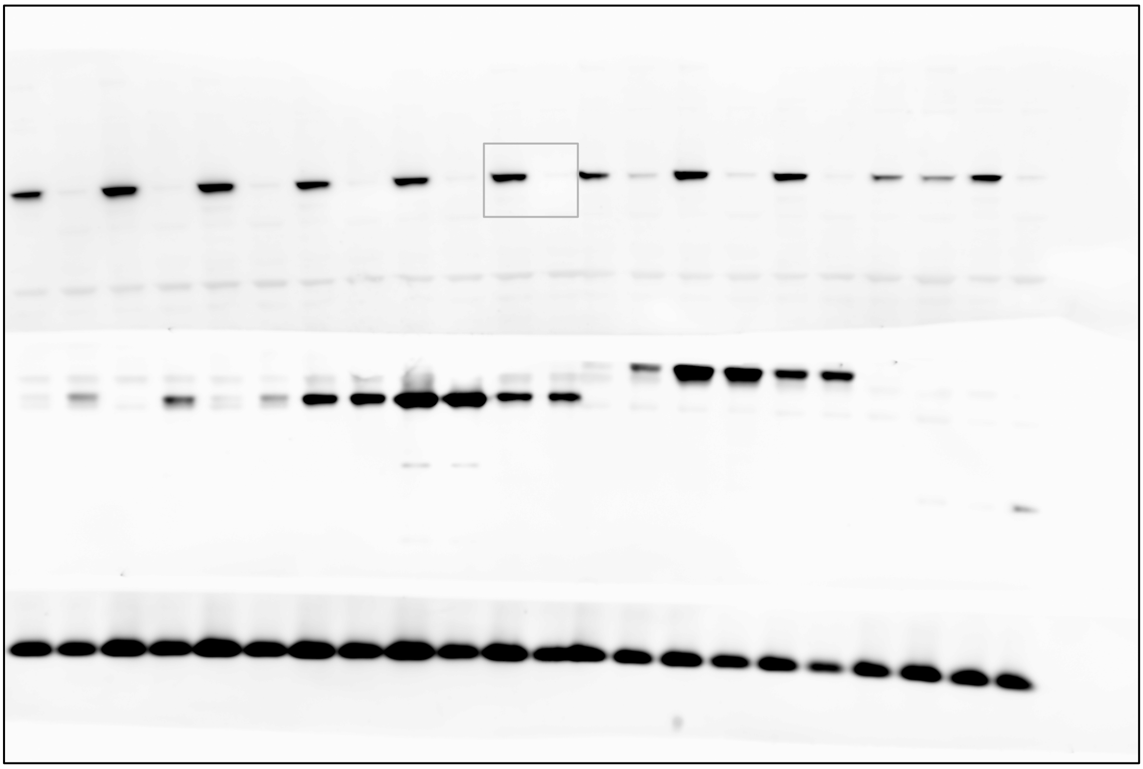

Lon

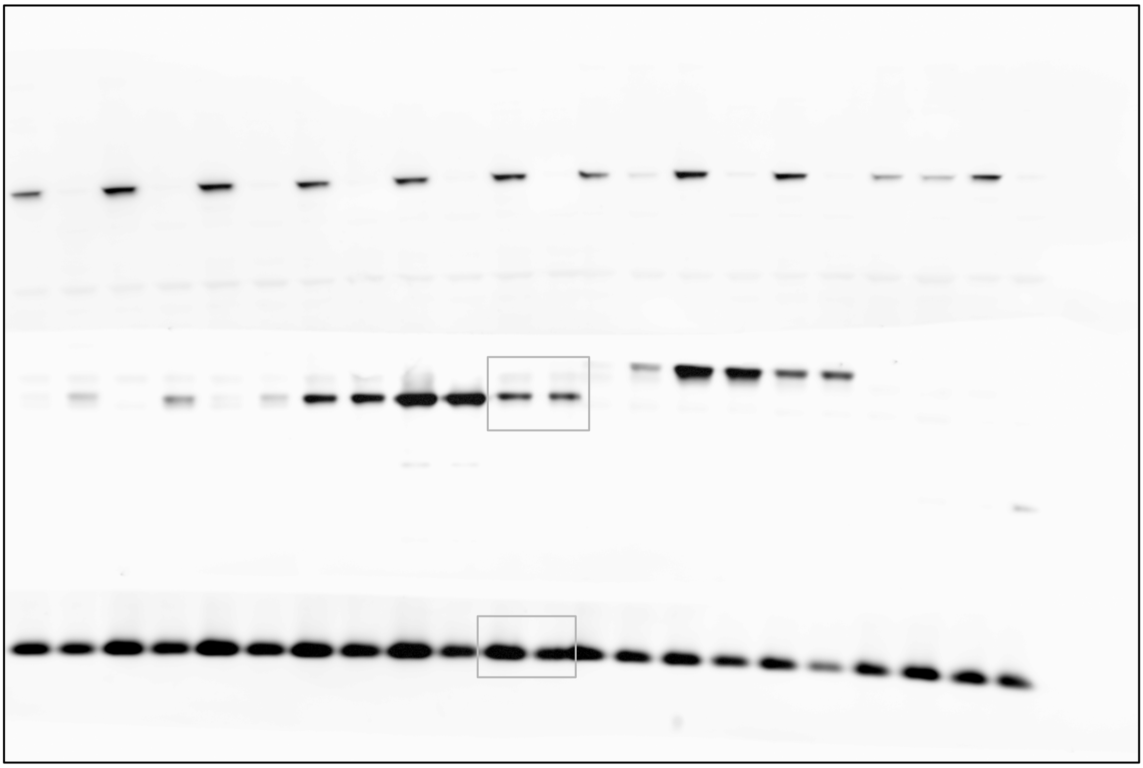

FLAG

LC

Fig. S10D (MPN201_Ct_FLAG mut2)

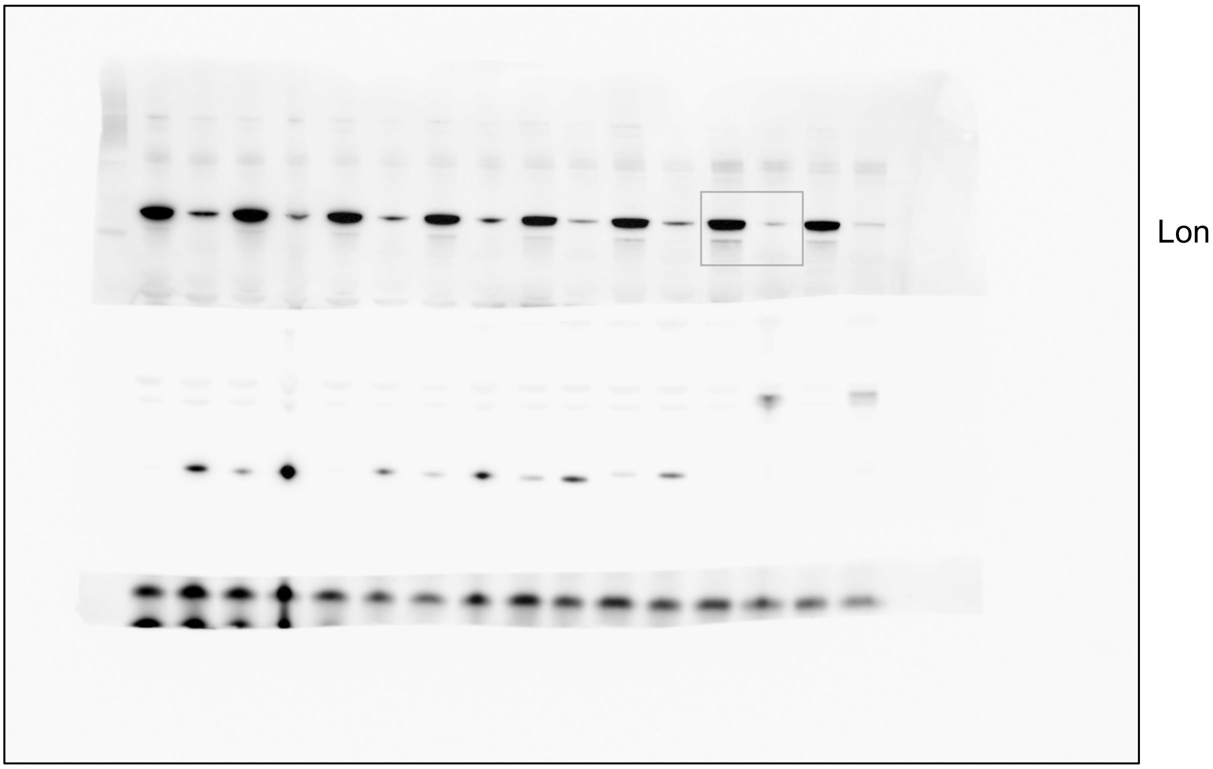

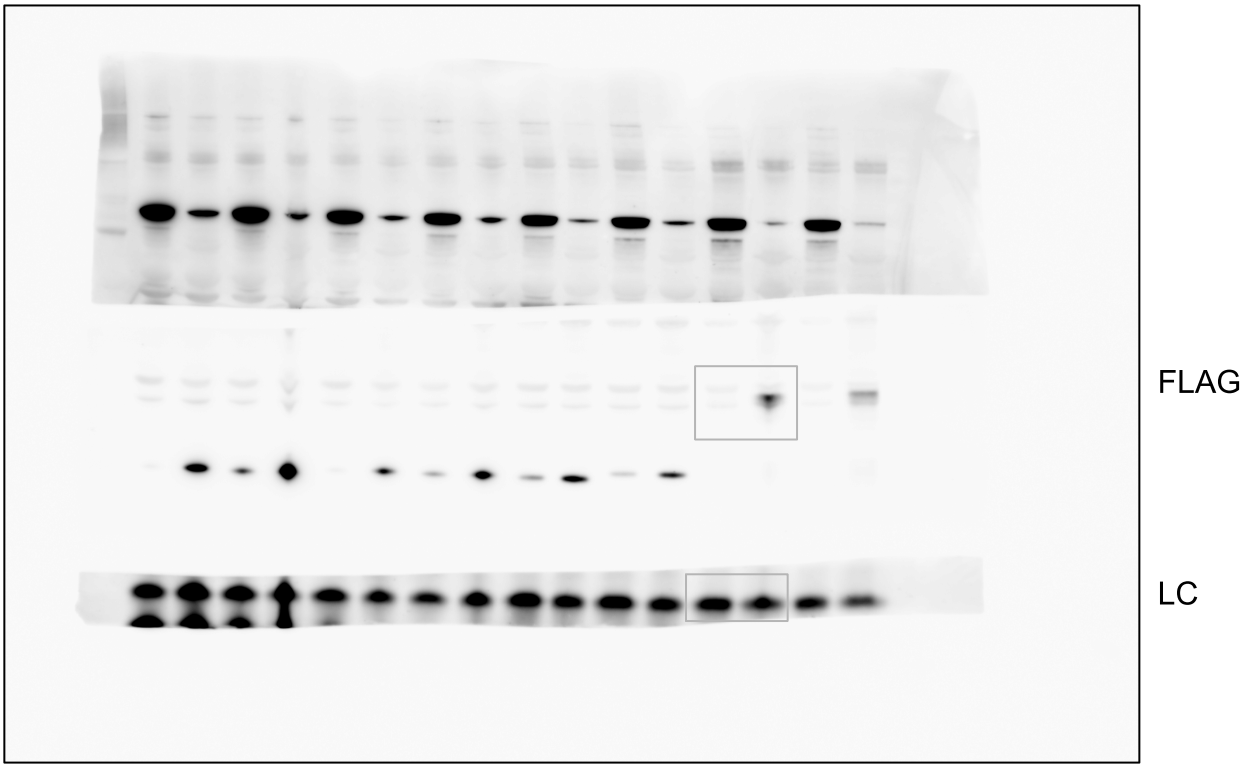

Fig. S10D (MPN201_Ct_FLAG mut3)

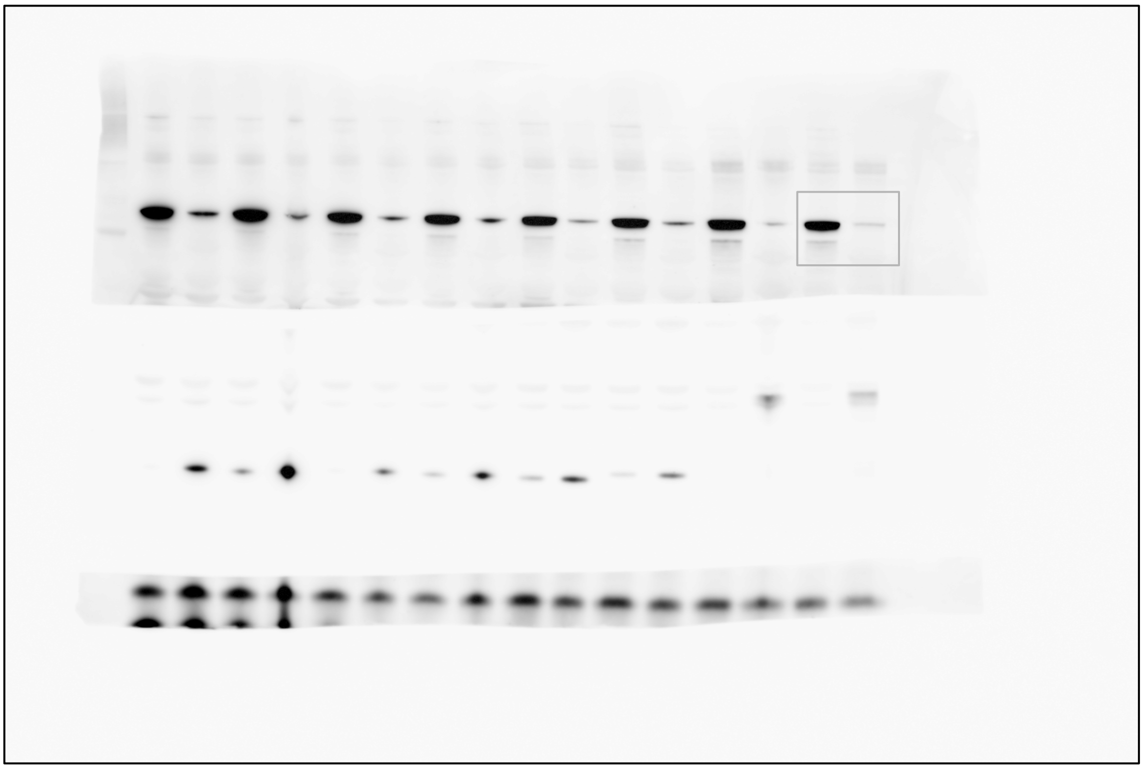

Lon

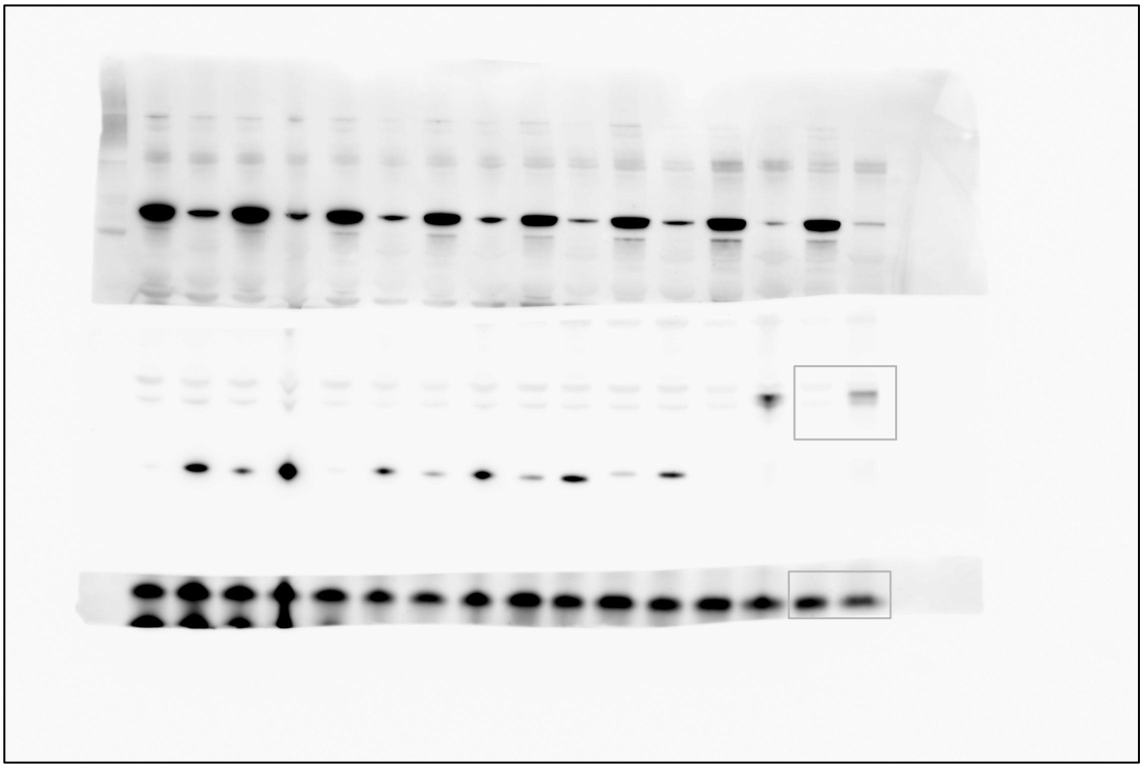

FLAG

LC

Fig. S10D (MPN201_Ct_FLAG mut4)

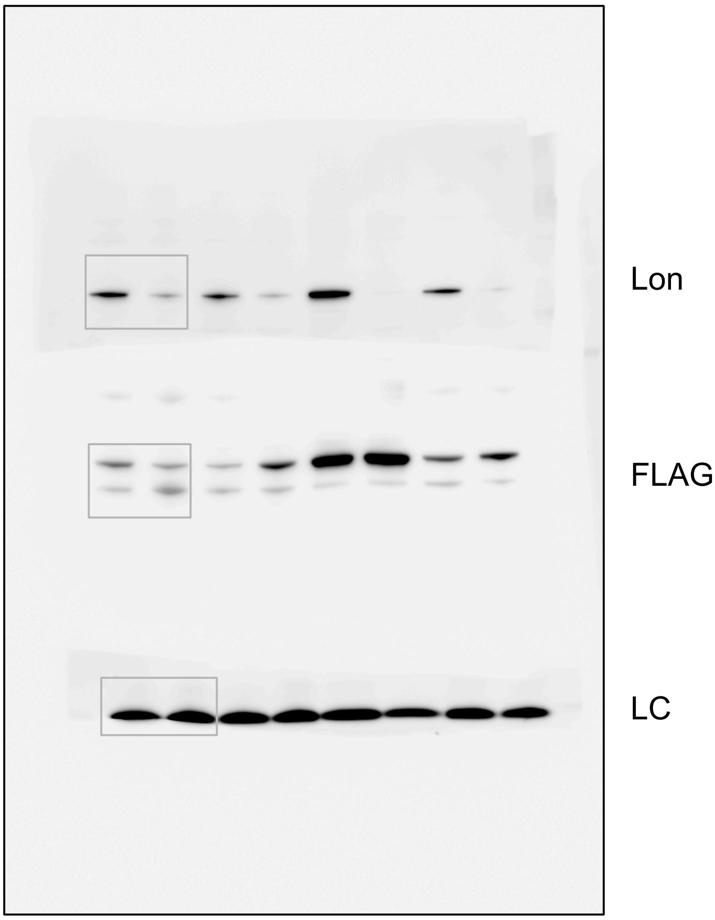

Supplement: Supplementary file 7 — Source Data for Appendix [file MSB-16-e9530-s007.zip › MSB-20-9530RR-Appendix_Figure_S10_Source_Data-sd.pdf]
