# Supplementary material for: Protein quality control and regulated proteolysis in the genome‐reduced organism Mycoplasma pneumoniae
Source: Mol Syst Biol. 2020 Dec 15;16(12):e9530. doi: 10.15252/msb.20209530 (PMC7737663; doi:10.15252/msb.20209530)

Appendix Figure S6

Fig. S6A (FtsA_MPN316)

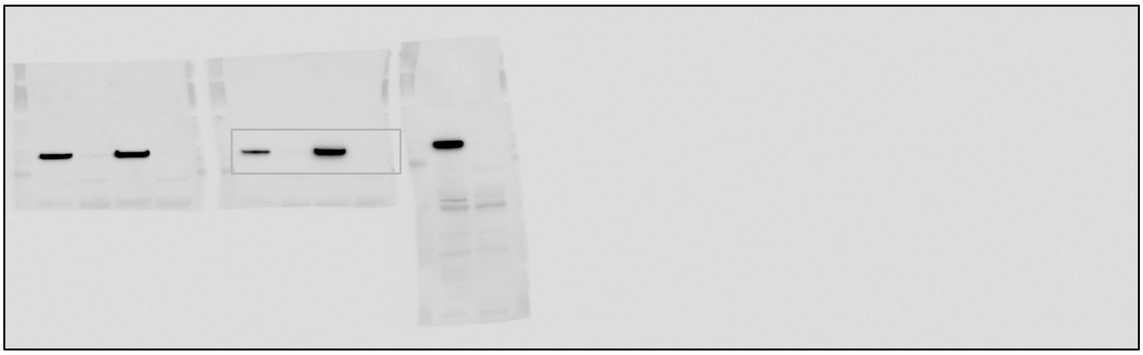

Lon

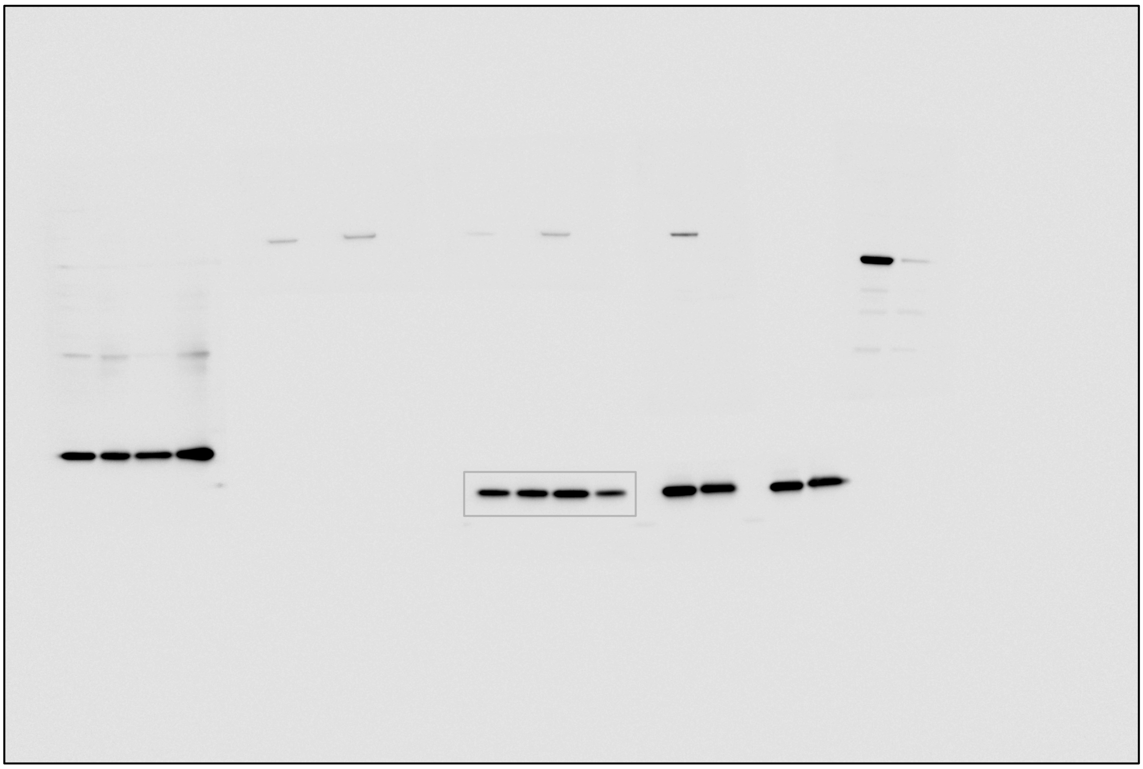

LC

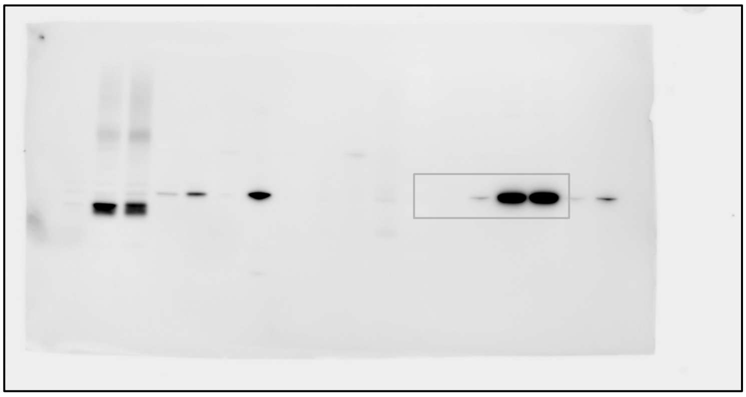

FLAG

Fig. S6A (FtsZ_MPN317)

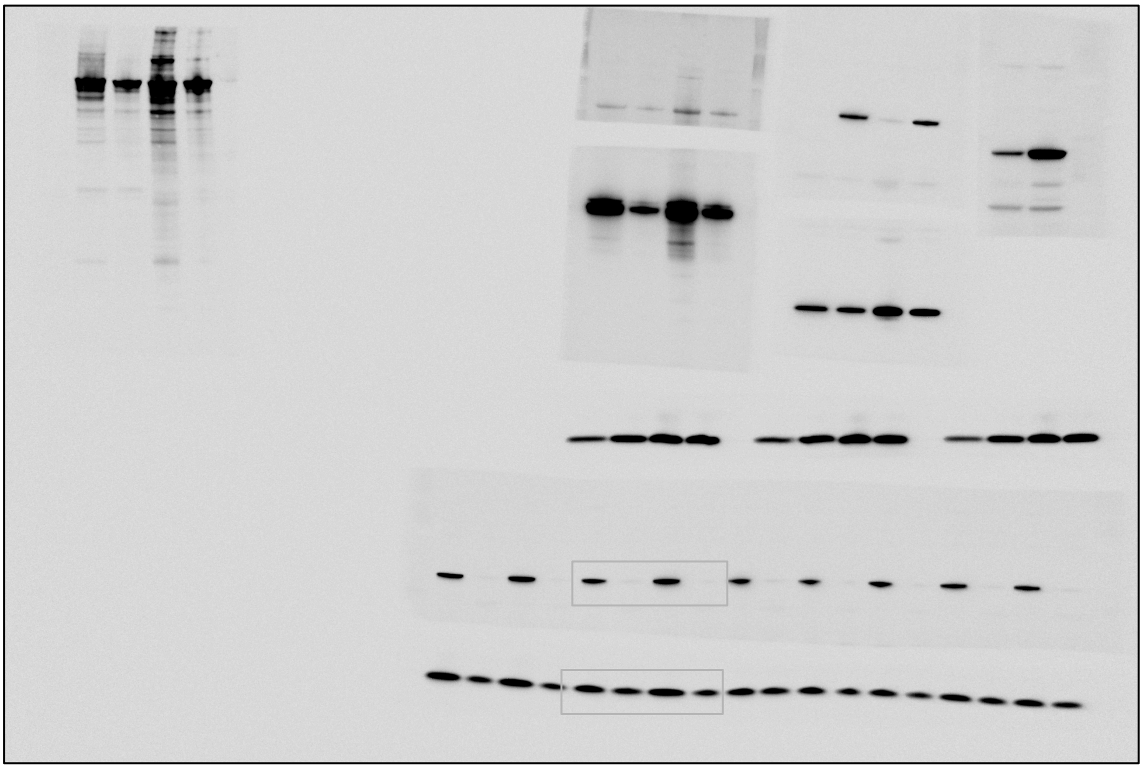

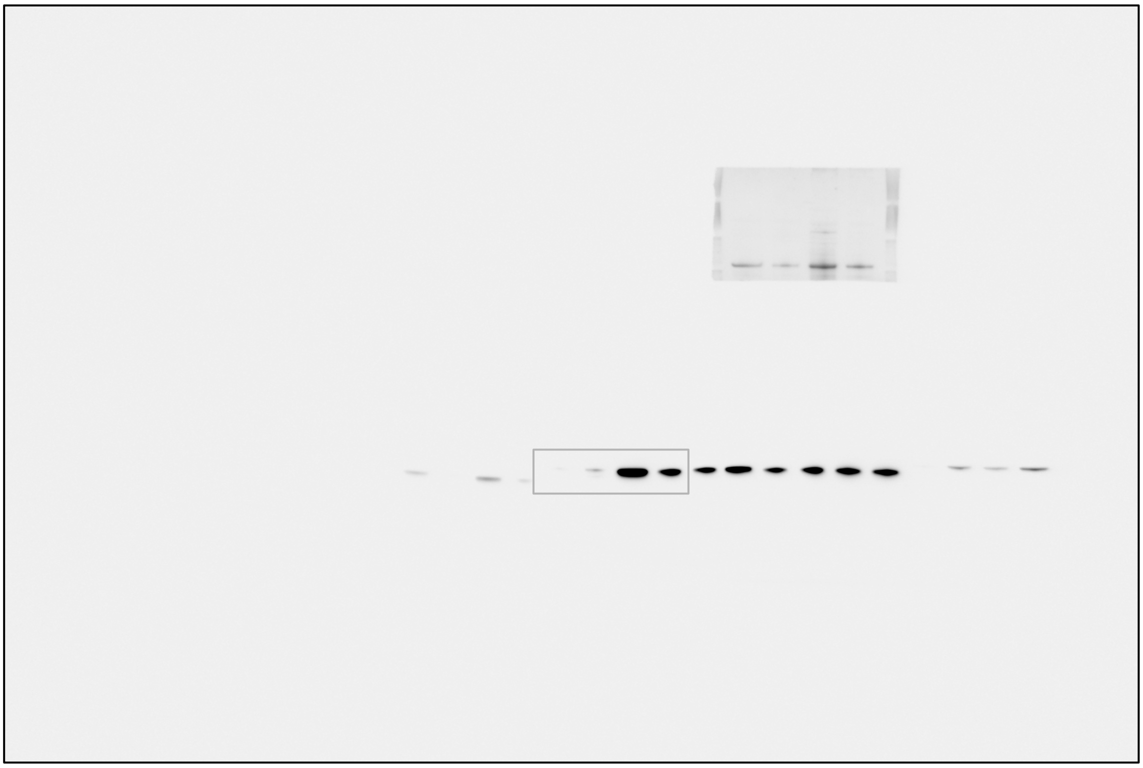

Fig. S6A (DnaB_MPN525)

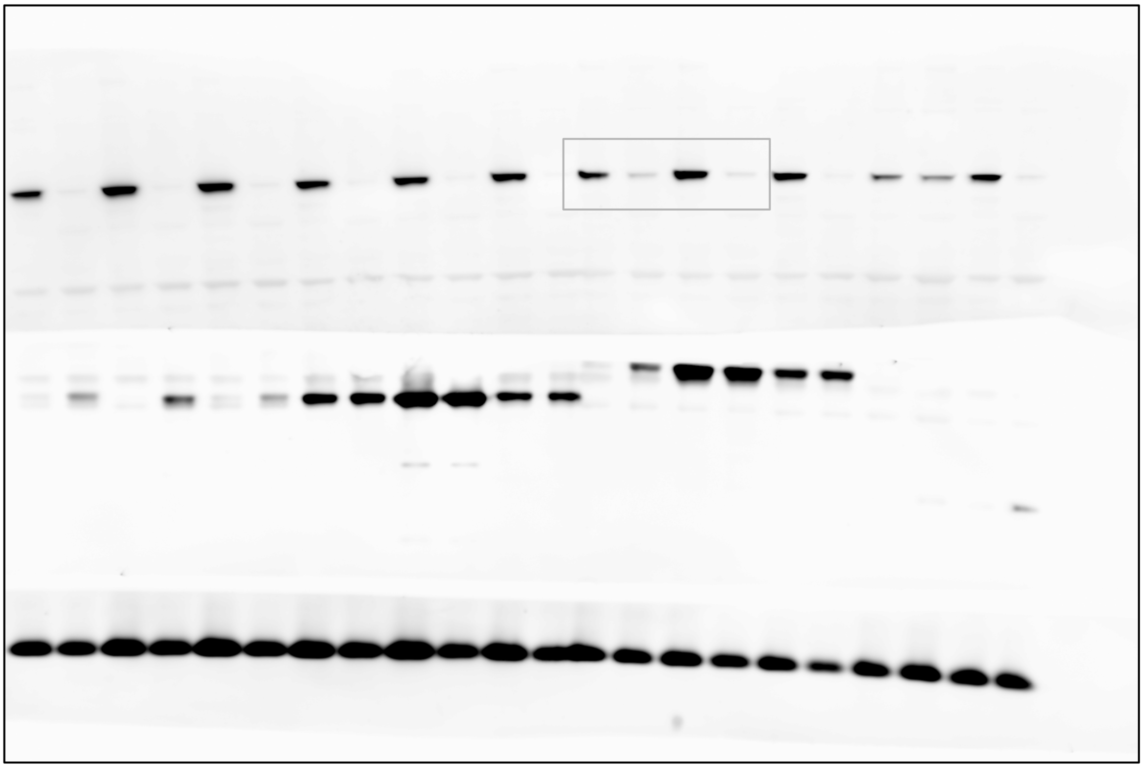

Lon

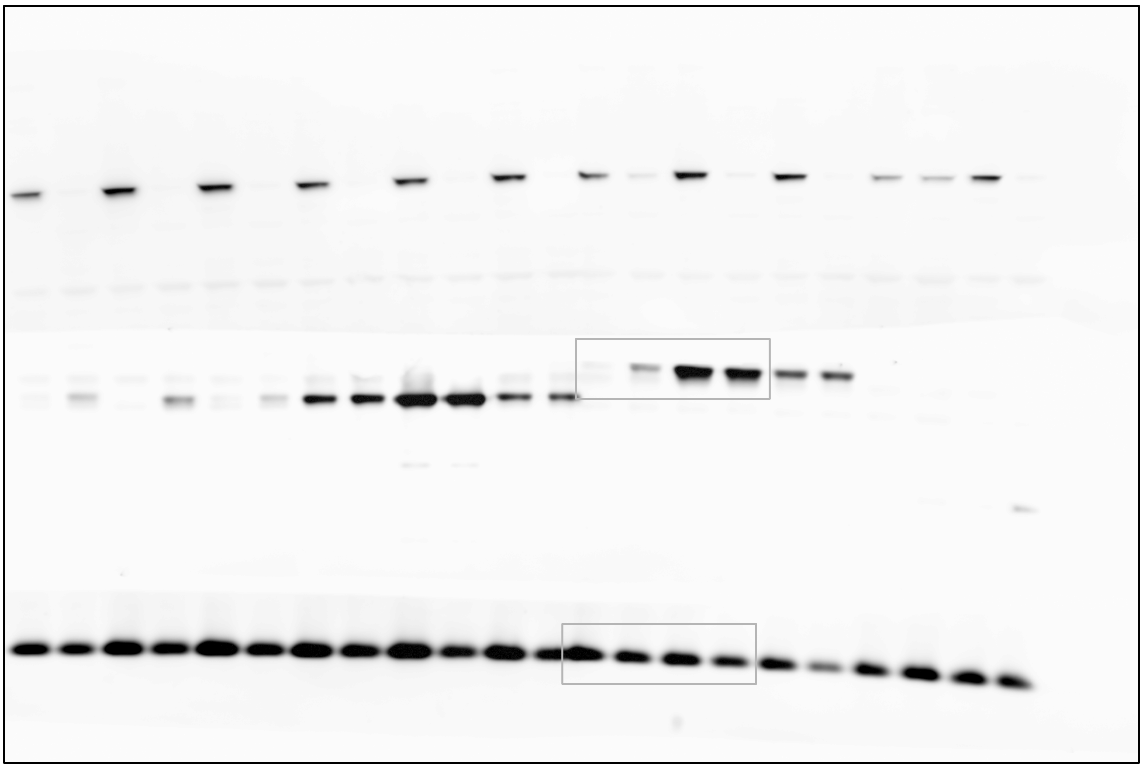

FLAG

LC

Fig. S6A (HsdS_MPN201)

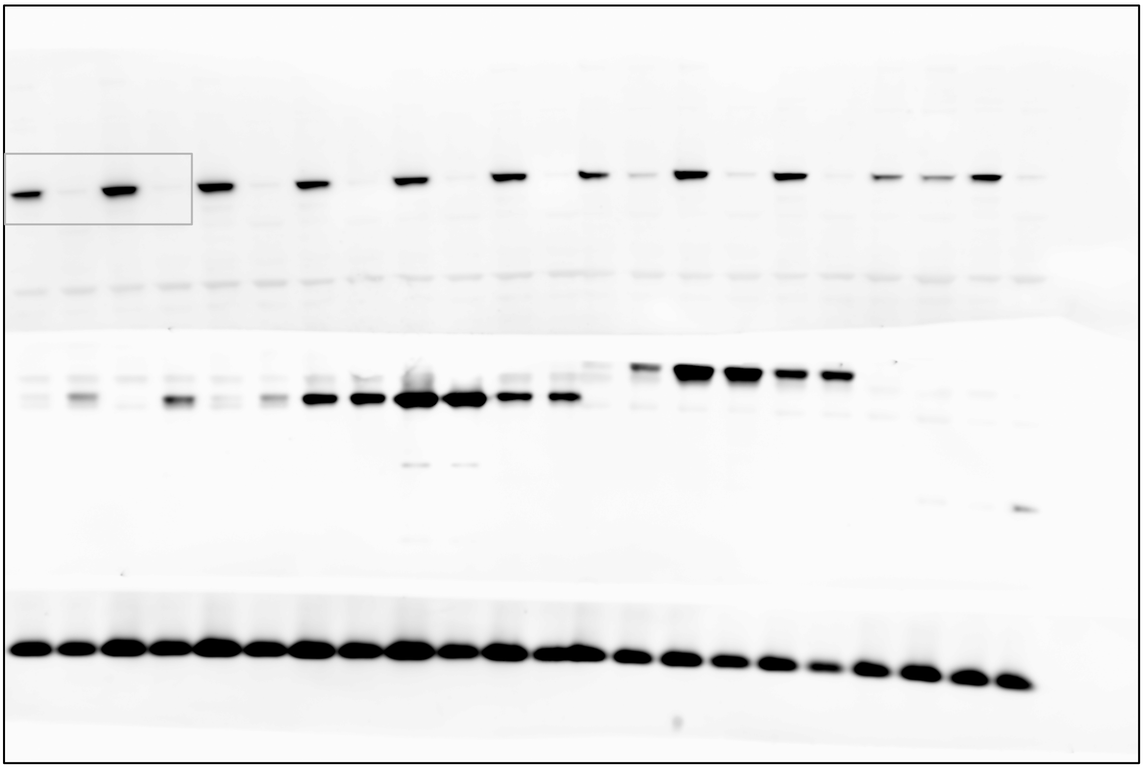

Lon

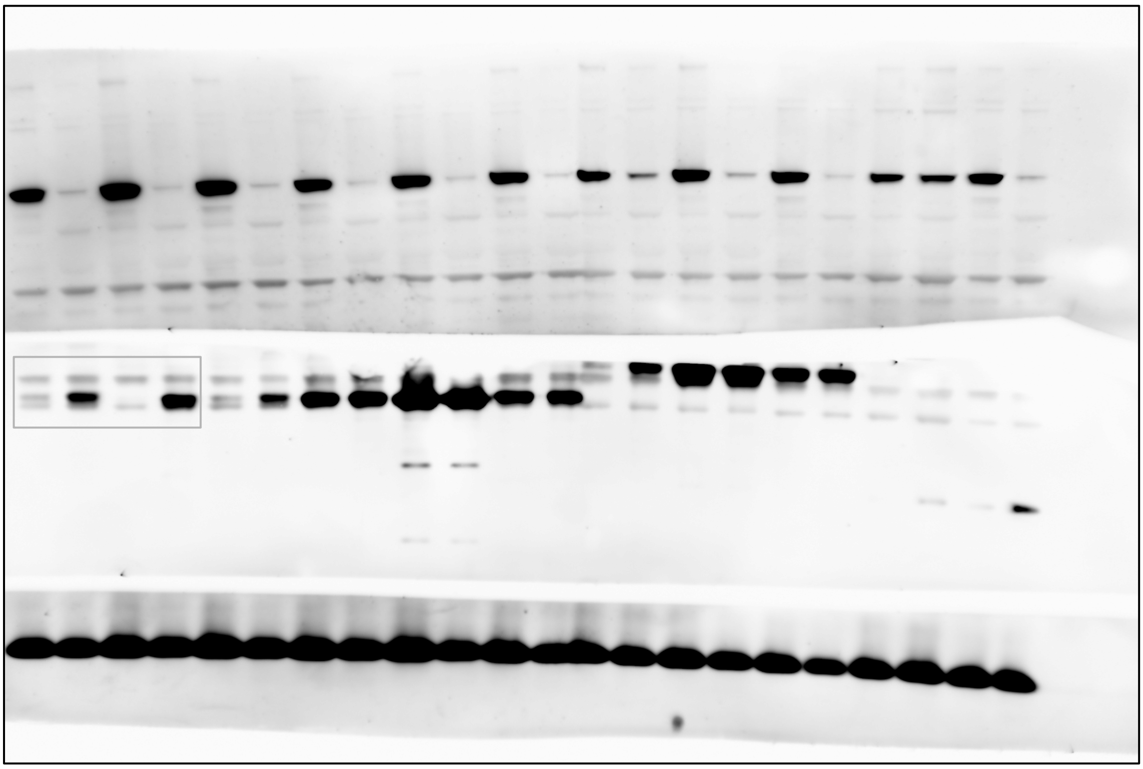

FLAG

Fig. S6A (HsdS_MPN201). cont

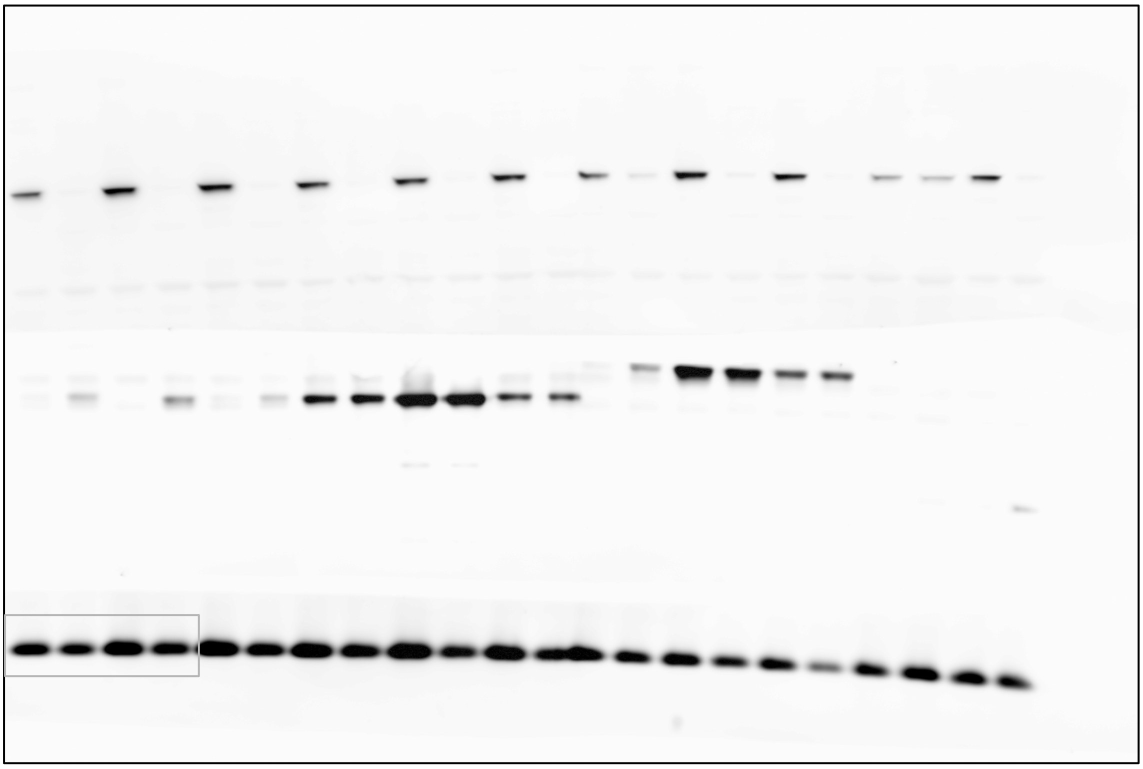

Fig. S6A (HsdS_MPN638)

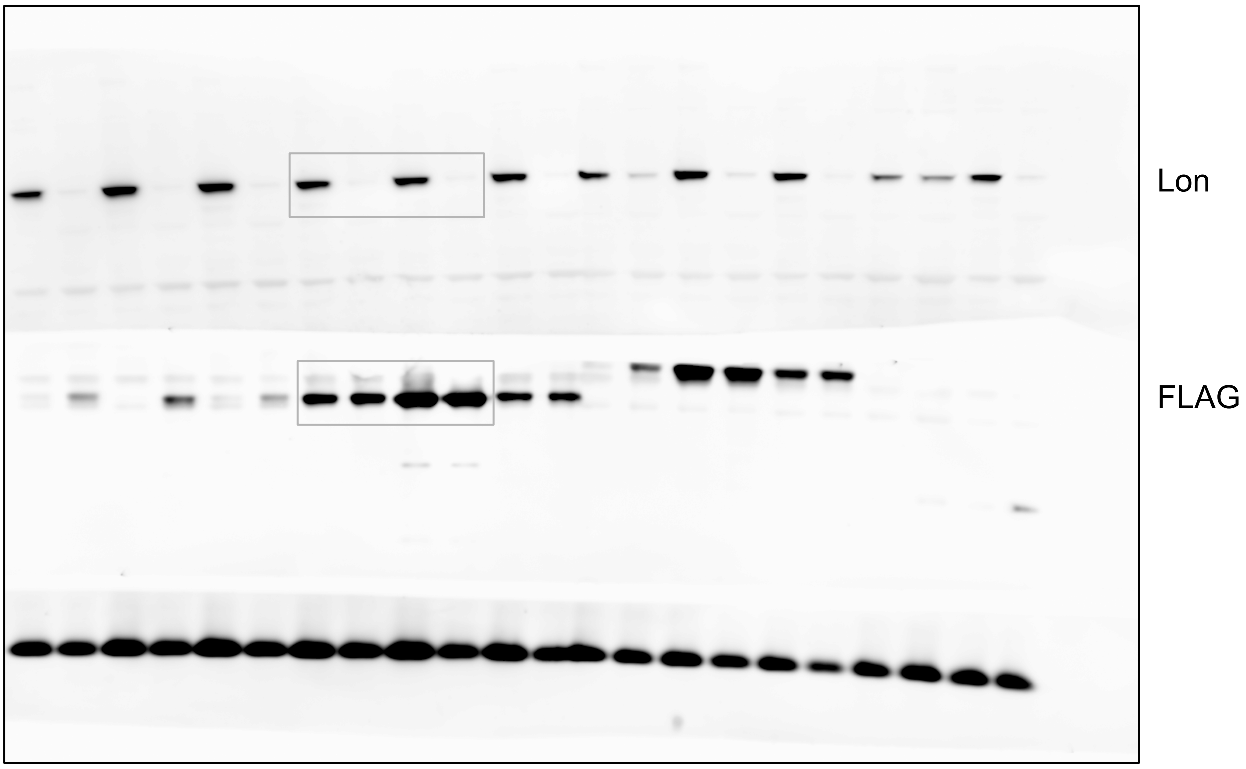

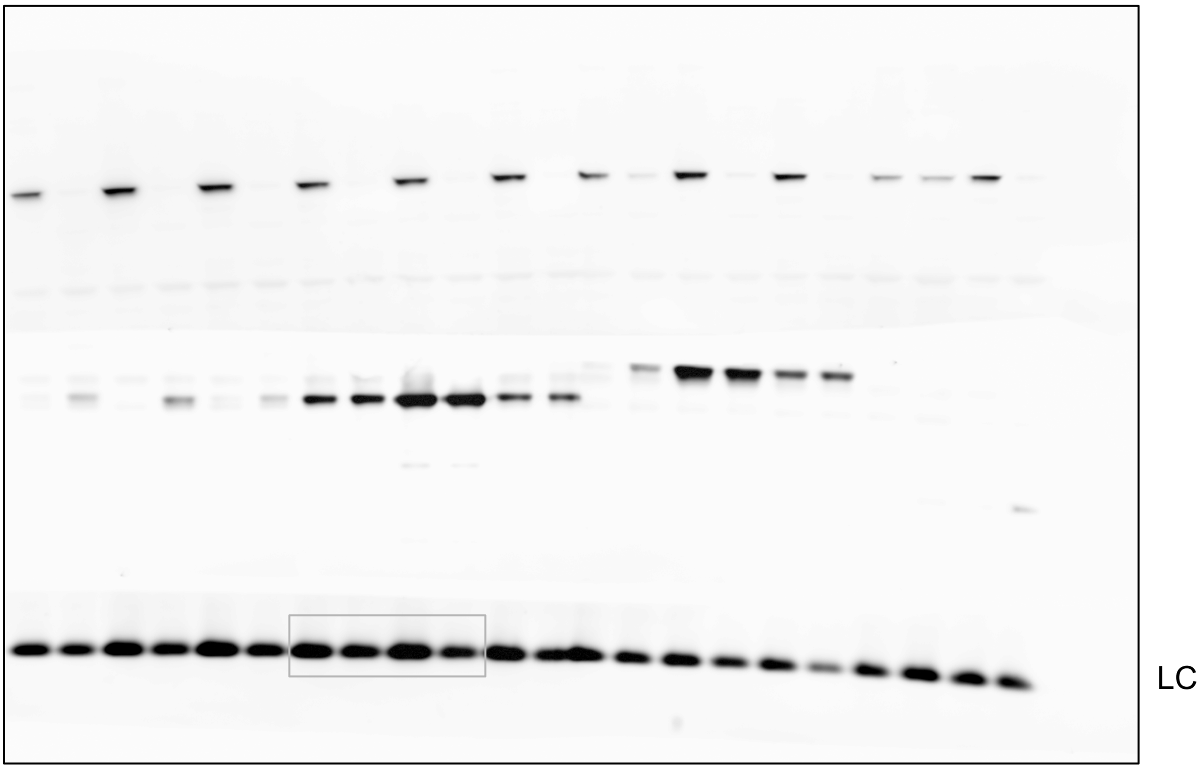

Fig. S6A (Nt_ArcA_MPN304)

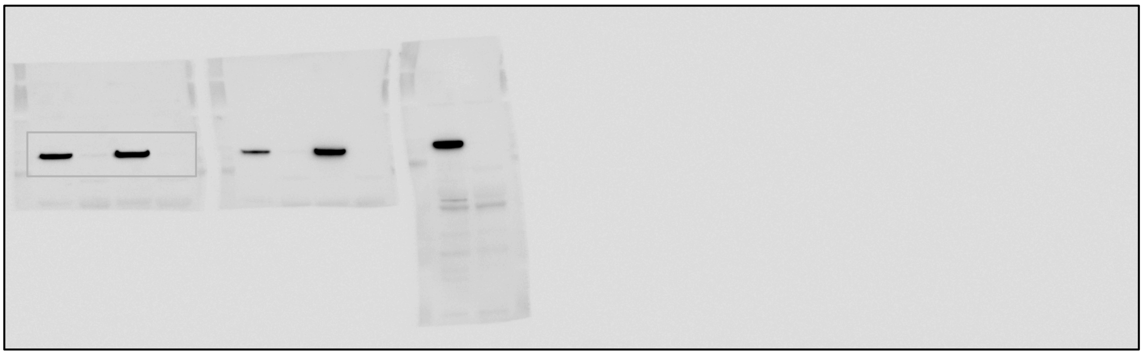

Lon

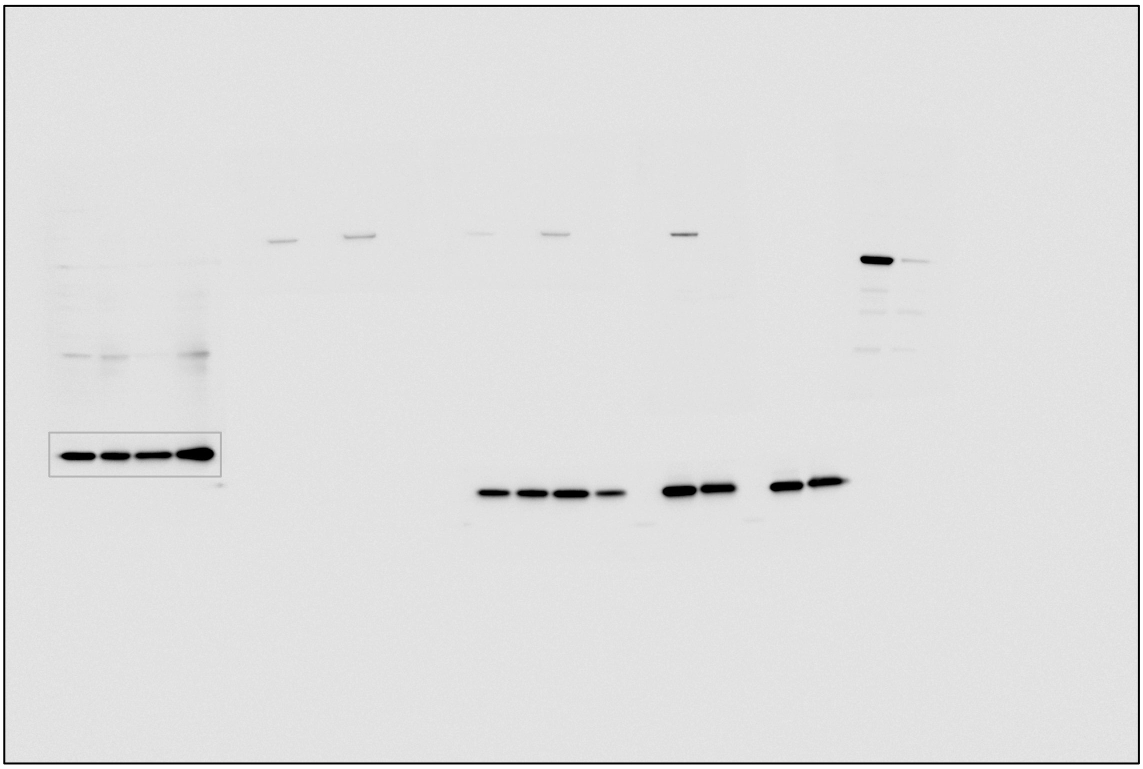

LC

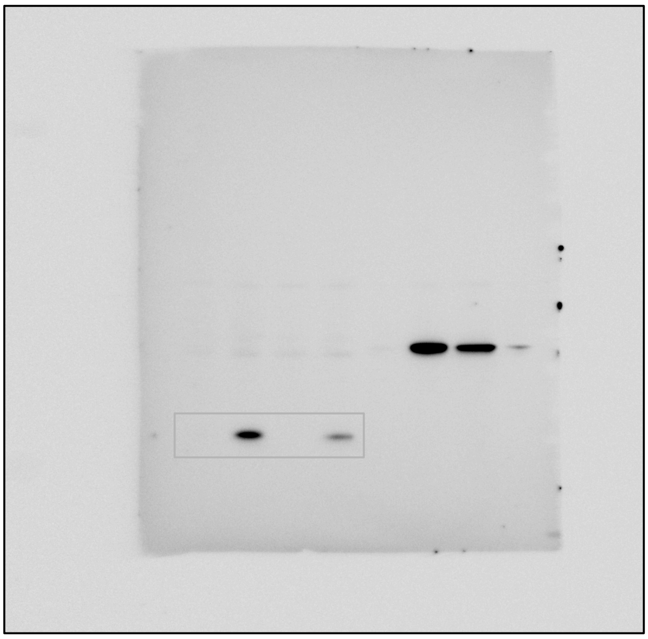

FLAG

Fig. S6B (MPN207a)

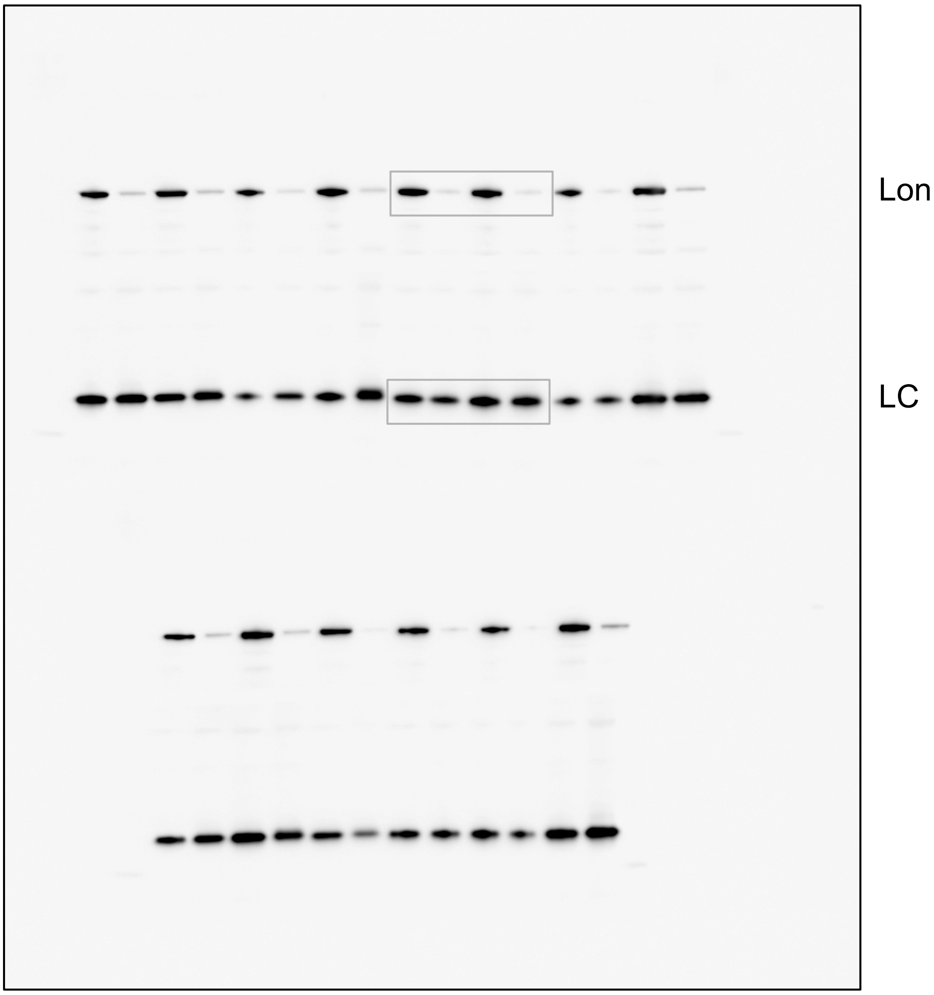

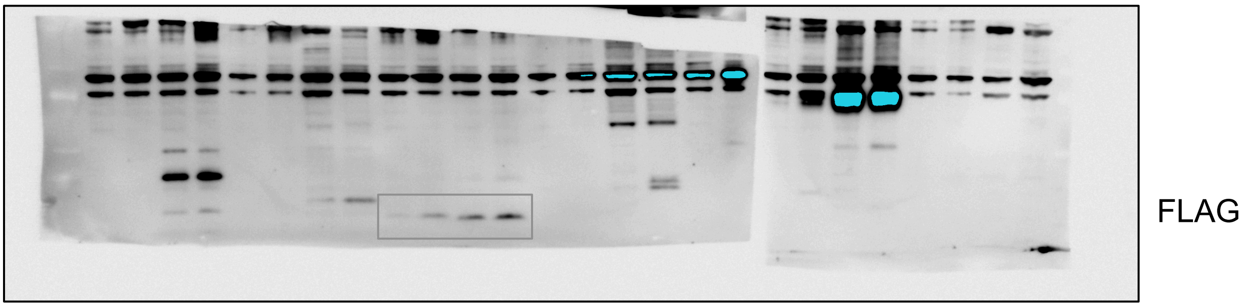

Fig. S6B (MPN308)

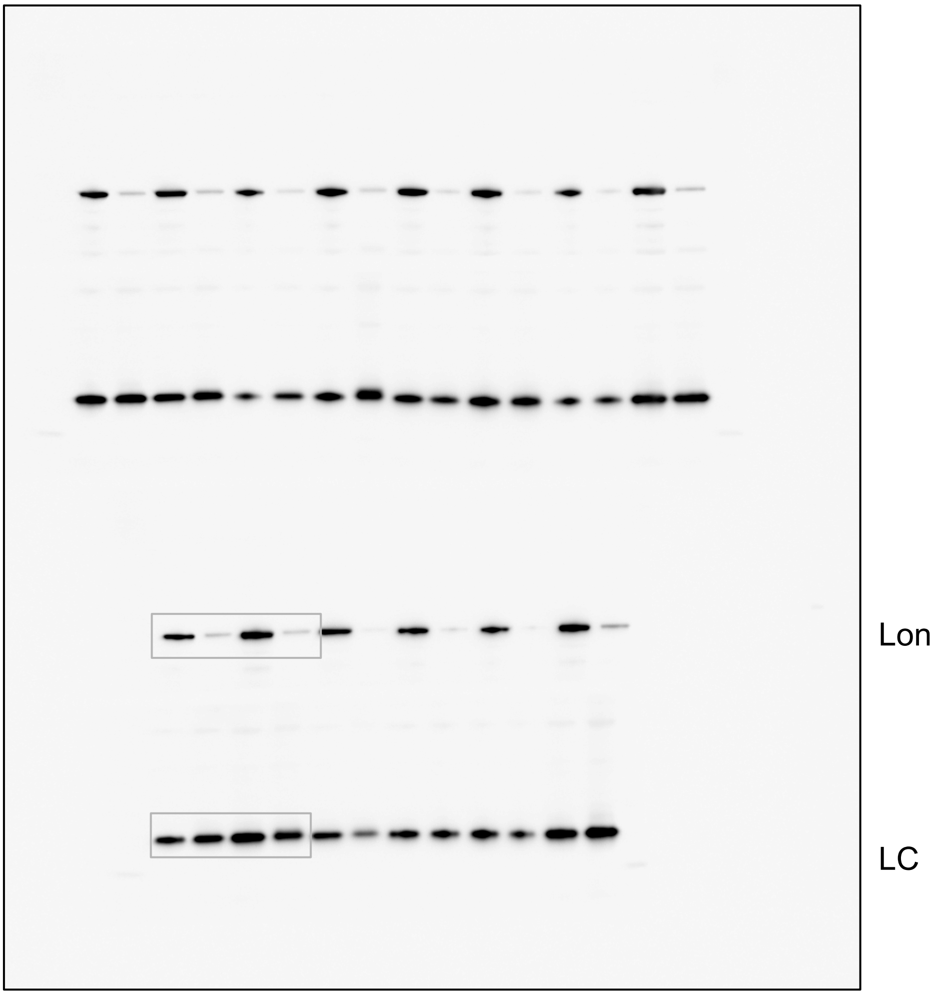

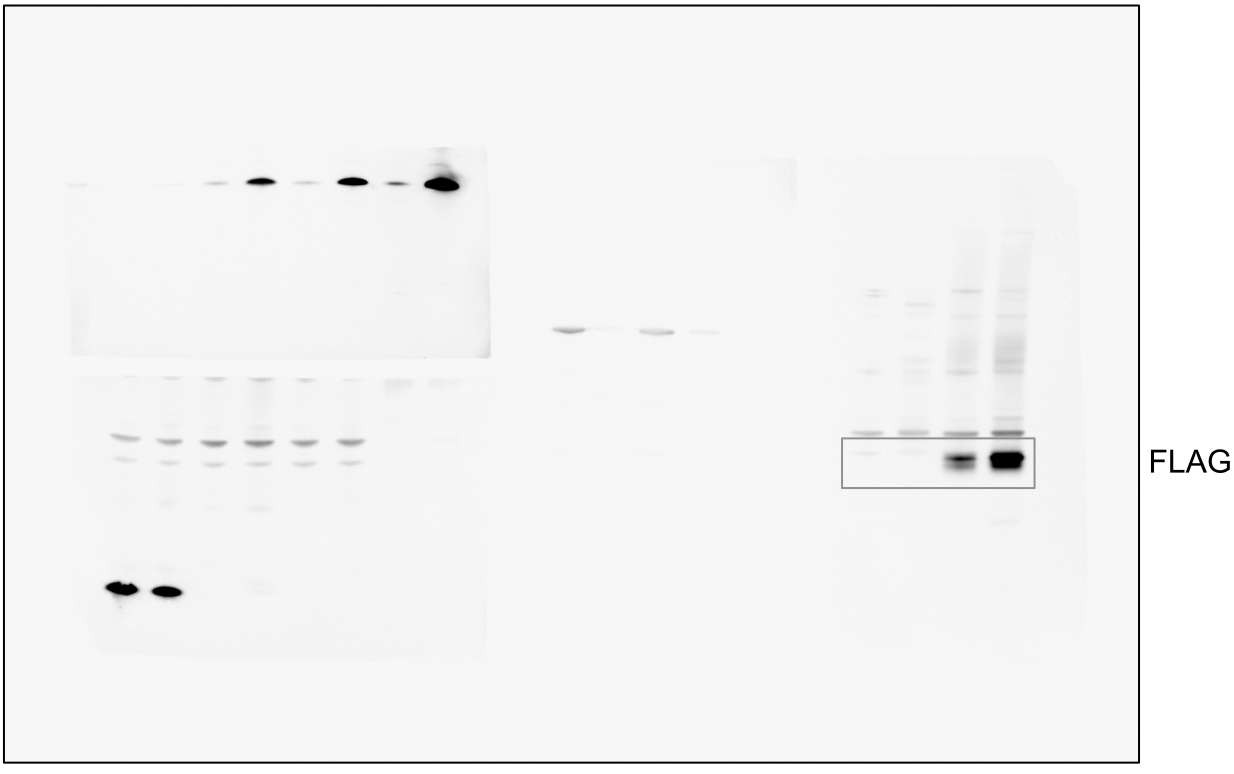

Fig. S6B (MPN449)

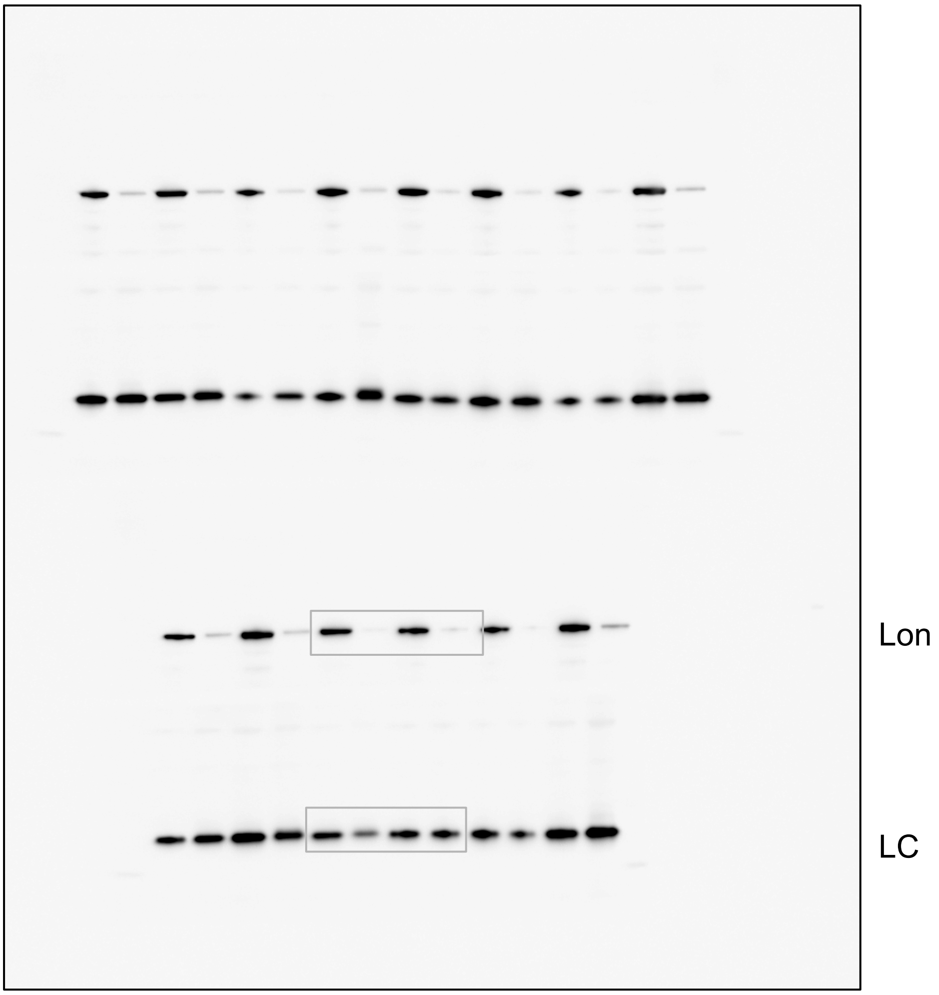

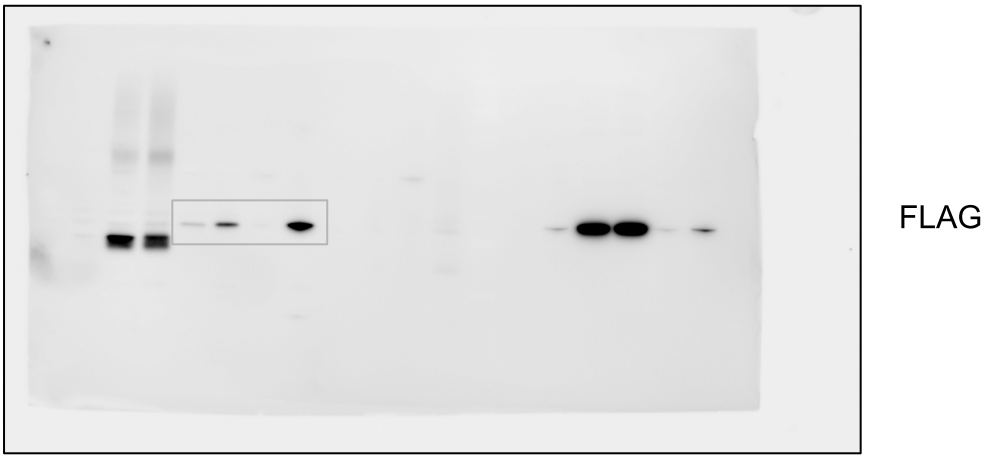

Supplement: Supplementary file 7 — Source Data for Appendix [file MSB-16-e9530-s007.zip › MSB-20-9530RR-Appendix_Figure_S6_Source_Data-sd.pdf]
