# Supplementary figures and images for: Protein quality control and regulated proteolysis in the genome‐reduced organism Mycoplasma pneumoniae
Source: Mol Syst Biol. 2020 Dec 15;16(12):e9530. doi: 10.15252/msb.20209530 (PMC7737663; doi:10.15252/msb.20209530)

## Appendix Figure S1

$\Delta$ IndLon strain

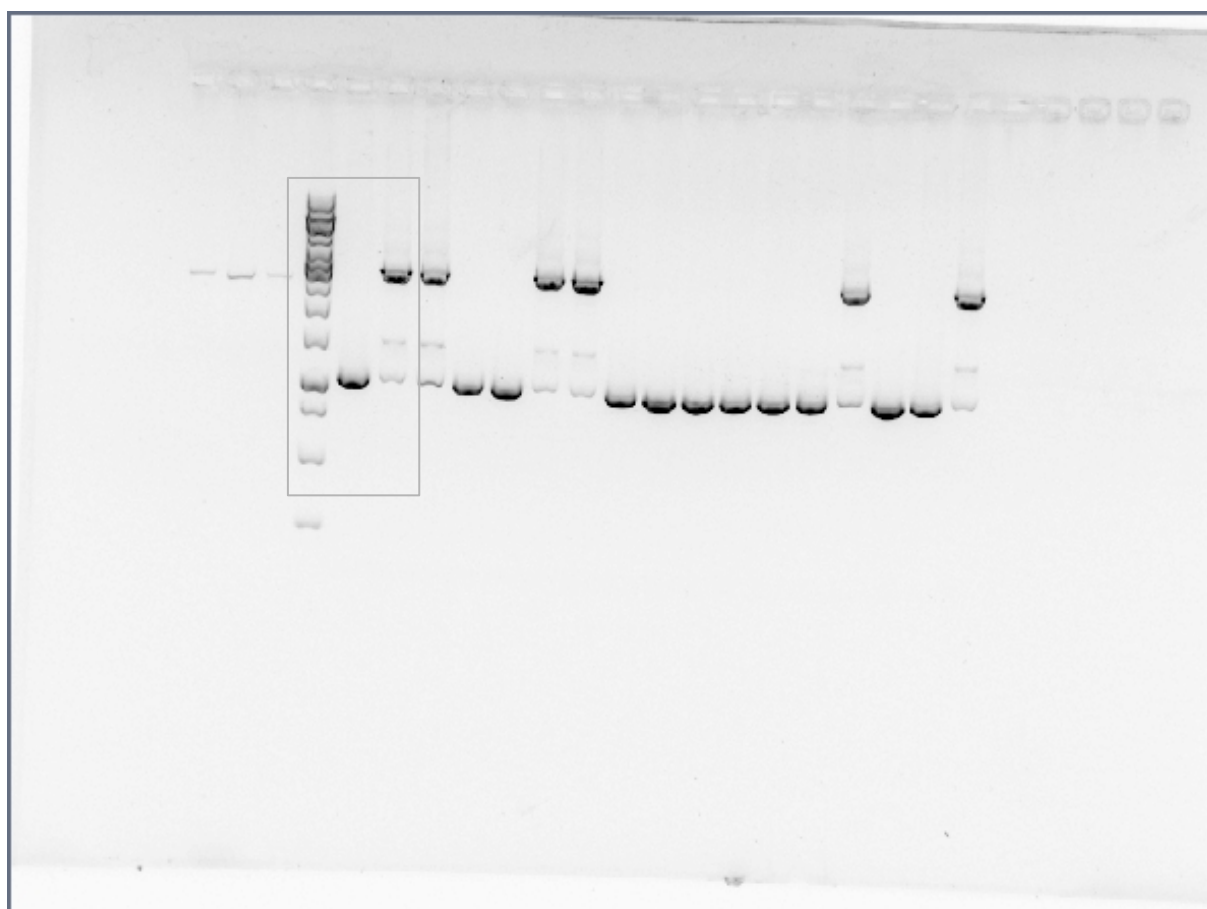

$\Delta$ IndFtsH strain

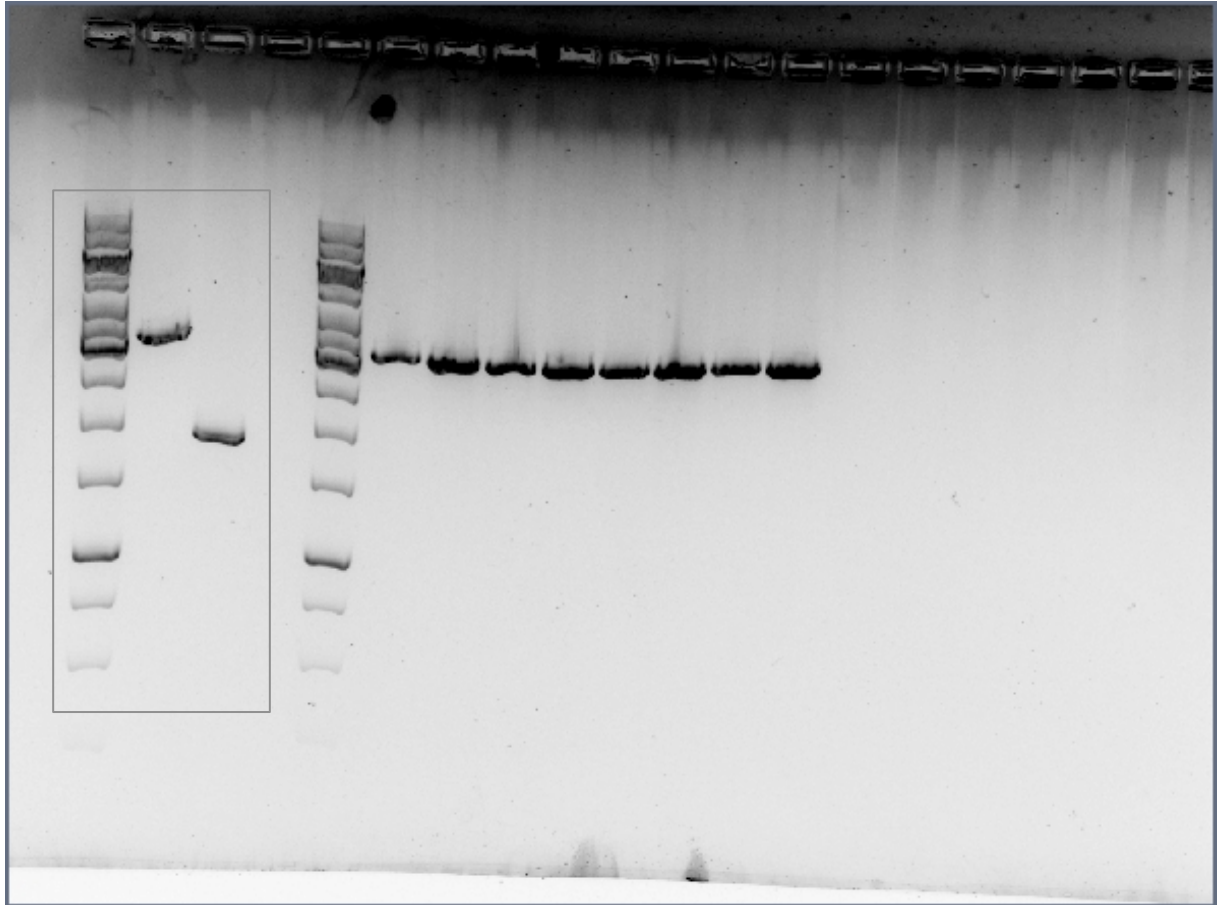

$\Delta$ IndLon\_FtsH strain

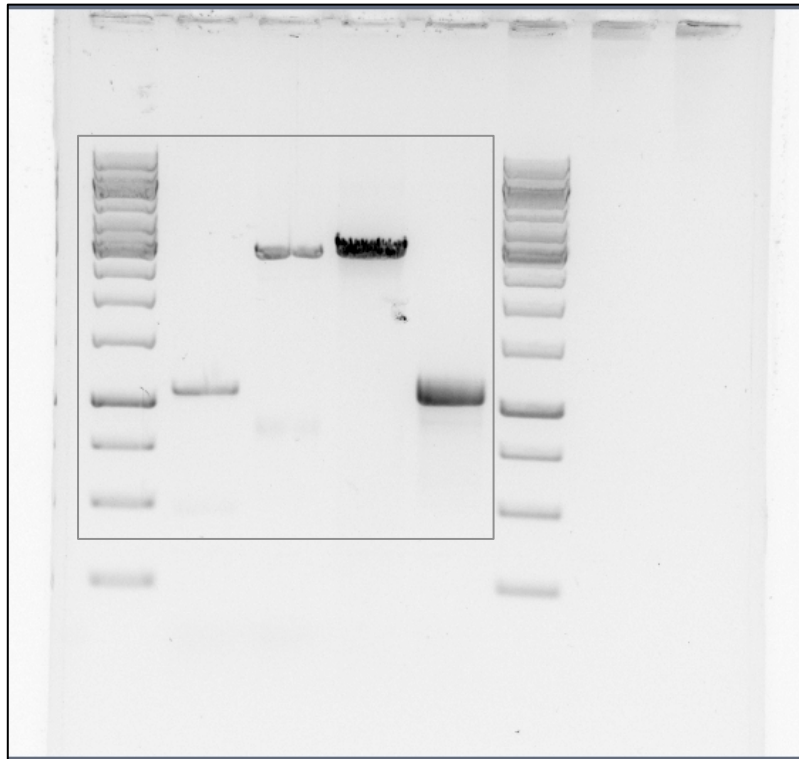

Supplement: Supplementary file 7 — Source Data for Appendix [file MSB-16-e9530-s007.zip › MSB-20-9530RR-Appendix_Figure_S1_Source_Data-sd.pdf]
